# An ancient neurotrophin receptor code; a single Runx/Cbfβ complex determines somatosensory neuron fate specification in zebrafish

**DOI:** 10.1371/journal.pgen.1006884

**Published:** 2017-07-14

**Authors:** Philia Gau, Andrew Curtright, Logan Condon, David W. Raible, Ajay Dhaka

**Affiliations:** 1 Department of Biological Structure, University of Washington, Seattle, Washington, United States of America; 2 Neuroscience Graduate Program, University of Washington, Seattle, Washington, United States of America; University of Pennsylvania, UNITED STATES

## Abstract

In terrestrial vertebrates such as birds and mammals, neurotrophin receptor expression is considered fundamental for the specification of distinct somatosensory neuron types where TrkA, TrkB and TrkC specify nociceptors, mechanoceptors and proprioceptors/mechanoceptors, respectively. In turn, Runx transcription factors promote neuronal fate specification by regulating neurotrophin receptor and sensory receptor expression where Runx1 mediates TrkA+ nociceptor diversification while Runx3 promotes a TrkC+ proprioceptive/mechanoceptive fate. Here, we report in zebrafish larvae that orthologs of the neurotrophin receptors in contrast to terrestrial vertebrates mark overlapping and distinct subsets of nociceptors suggesting that TrkA, TrkB and TrkC do not intrinsically promote nociceptor, mechanoceptor and proprioceptor/mechanoceptor neuronal fates, respectively. While we find that zebrafish Runx3 regulates nociceptors in contrast to terrestrial vertebrates, it shares a conserved regulatory mechanism found in terrestrial vertebrate proprioceptors/mechanoceptors in which it promotes TrkC expression and suppresses TrkB expression. We find that Cbfβ, which enhances Runx protein stability and affinity for DNA, serves as an obligate cofactor for Runx in neuronal fate determination. High levels of Runx can compensate for the loss of Cbfβ, indicating that in this context Cbfβ serves solely as a signal amplifier of Runx activity. Our data suggests an alteration/expansion of the neurotrophin receptor code of sensory neurons between larval teleost fish and terrestrial vertebrates, while the essential roles of Runx/Cbfβ in sensory neuron cell fate determination while also expanded are conserved.

## Author summary

Our perception of the external world comes from our senses. Often overlooked the skin is our largest sensory organ. Specialized neurons located in the dorsal root ganglion (DRG), which innervate the body, and trigeminal ganglion (TG), which innervate the face, sense the somatosensory perceptions: light touch, temperature, pain (nociceptors) and muscle/limb position (proprioception) via nerve endings that project to the skin. These neurons receive and relay information from these diverse stimuli through distinct subclasses of neurons. Since these neurons arise from common lineages, they provide an excellent system to study how neurons develop and diversify into different subtypes. Runx transcription factors have been shown in terrestrial vertebrates (birds and mammals) to be instrumental in specifying nociceptor and proprioceptor populations by regulating the expression of a class of genes that code for the neurotrophin receptors, which are thought to be essential for specifying these neuronal fates. In our study we show that mechanisms by which Runx transcription factors regulate neurotrophin receptor expression are conserved between zebrafish and terrestrial vertebrates, yet the type of neuron specified by these genes are different such that in zebrafish the neurotrophin receptor TrkC is expressed in a nociceptor lineage instead of the proprioceptor/mechanoreceptor lineage as in terrestrial vertebrates. These data demonstrate that the specification of neuronal lineages is not fundamental to a given neurotrophin receptor but has adapted and evolved from the time fish and terrestrial vertebrates diverged 350 million years ago. Furthermore we show in fish that zebrafish Runx3 has properties that are divided between Runx1 and Runx3 in terrestrial vertebrates. Finally we show that the Runx co-factor Cbfβ is essential for its function, but the high level of Runx3 expression can overcome the loss of Cbfβ, demonstrating that Cbfβ in this context serves solely as a signal amplifier of Runx3 activity.

## Introduction

Sensory neurons of the dorsal root ganglia (DRG) and trigeminal ganglia (TG) of terrestrial vertebrates convey somatosensory information from the body and face, respectively. Distinct but overlapping sensations, including touch, proprioception (body position), and nociception (pain) are perceived by different sensory neuron populations [1]. How these distinct neurons acquire their functional properties during development is an important question in understanding the assembly of the somatosensory circuits and may shed light on how these properties change in pathogenic states such as chronic pain.

A consensus model has emerged for how distinct sensory neuron populations develop in mammalian embryos. After their initial establishment from placodal and neural crest origins, sensory neurons become specialized into distinct populations. The development and survival of these populations are controlled by tropomyosin-receptor kinase (Trk) proteins that act as receptors for the neurotrophin family of growth factors. In the DRG, the three primary classes of sensory neurons are marked by distinct expression of neurotrophin receptors: nociceptors including thermoceptors and pruriceptors express TrkA, mechanoreceptors express TrkB and proprioceptors express TrkC [1]. In the TG, TrkC is primarily associated with mechanoreceptors that do not coexpress TrkB [2]. However, it appears that in early zebrafish development neurotrophin receptors may label different subset of somatosensory neurons [3].

The specification of sensory neuron subtypes is under transcription factor (TF) control. In particular, the Runt domain (Runx) TFs have been shown to be key regulators of somatosensory cell fate in terrestrial vertebrates. In the DRG, Runx3 is critical for the development of proprioceptors where it acts to suppress TrkB expression and promote TrkC expression, while in the TG Runx3 is required for the specification of TrkC-expressing mechanoceptors that innervate Merkel cells [2,4–6]. Runx1, which is expressed in a largely distinct population of somatosensory neurons from Runx3, is required for the specification of nonpeptidergic nociceptors from TrkA+ precursors [7,8]. Runx1 is also required for the expression of numerous somatosensory receptors including TRPV1, TRPA1 and Piezo2 [7,8]. The transcription cofactor Core binding factor-beta (CBFβ) is required for Runx-mediated signaling where it functions to enhance Runx binding to DNA 5 to-10-fold and protect Runx from ubiquitin-mediated degradation [9–11]. Whether or not CBFβ plays a modular or obligate role in Runx-mediated somatosensory neuron specification is unknown.

In this study we characterize the Trk receptor code in developing zebrafish and examine the roles of Runx TFs in regulating Trk expression and sensory neuron specification. While it is difficult to make comparisons across species that develop along different timescales and environmental constraints/pressures (terrestrial vertebrates develop *in utero* or *in ovo*, while zebrafish develop as free-swimming larvae that are capable of perceiving and responding to external stimuli), we found striking conservation of function as well as substantial differences between these animals. In contrast to terrestrial vertebrates, we find that in larval zebrafish, TrkA, TrkB and TrkC label distinct and overlapping classes of nociceptors. By generating loss of function mutations in zebrafish, we find that Runx3 supports a hybrid of the independent functions Runx1 and Runx3 play in terrestrial vertebrates. We also illuminate the role Cbfβ plays in Runx mediated neuronal specification, showing that Cbfβ acts as a signal amplifier of Runx3 signaling whose loss can be overcome by excess Runx3 expression. These observations argue that while fish diverged from terrestrial vertebrates over 350 million years ago, and may have substantially different somatosensory requirements, core aspects of the molecular program regulating somatosensory neuron specification even in early larval zebrafish remain largely intact.

## Results

### Neurotrophin receptors mark nociceptors in larval zebrafish

**Fig 1:**
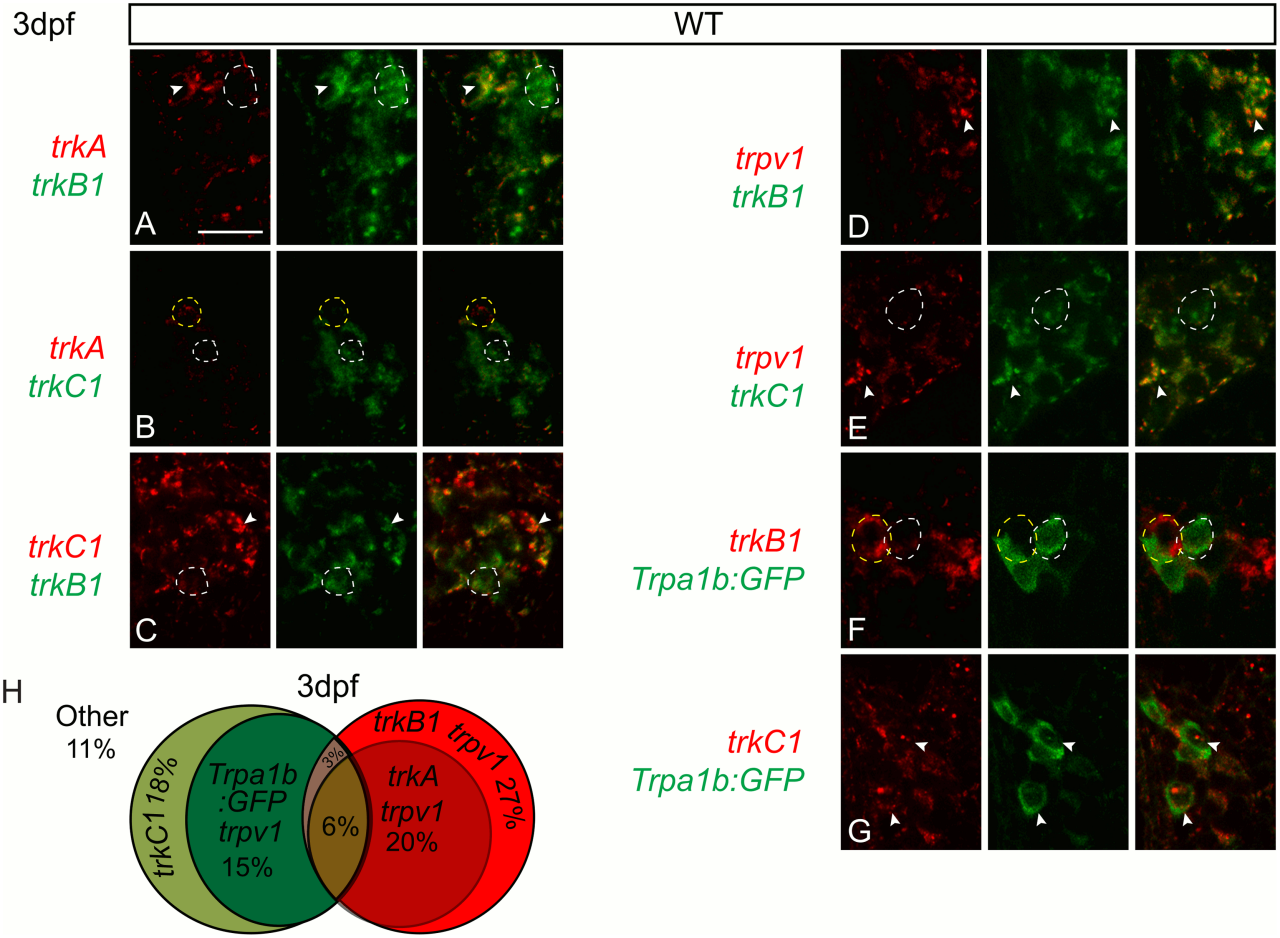
The zebrafish trigeminal ganglion contains many somatosensory subpopulations defined by the neurotrophin receptors and *trpv1* and *trpa1b* nociceptive ion channels. A-E, Optical sections of double fluorescent *in situ* hybridization for *trkA* (red) and *trkB1* (green) (A), *trkA* (red) and *trkB1* (green) (B), *trkA1* (red) and *trkC1* (green) (C), *trkC* (red) and *trkB1* (green) (D), *trpv1* (red) and *trkB1* (green) (E) and trpv1 (red) and trkC1 (green in wild-type fish at 3dpf. F-G, Optical sections of antibody staining of GFP (green) in conjunction with fluorescent *in situ* hybridization for *trkB1* (F) and *trkC1* (G) in *trpa1b*:*GFP* fish at 3dpf. H, Representations of 3dpf wild-type somatosensory subpopulations as a percent of the whole TG. Dashed white line outline green cells; Dashed yellow lines outline red cells; Arrowhead indicates double positive cells. Scale bar: 20μm. Embryos per condition (n = 3–4).

In the TG of larval zebrafish, we characterized the overlap of the neurotrophin receptor populations with each other and with the *trpv1* and *trpa1b* nociceptive ion channels, which respectively are required for larval zebrafish responses to noxious heat and the noxious chemicals, such as allyl isothiocyanate (AITC) [12]. We have previously shown as in mammals that neurons that express *trpa1b* are completely within the set that expresses *trpv1* [13]. In zebrafish at 3dpf, *trkC1* expression shows little overlap with *trkA* or *trkB1*, while *trkA* is expressed in a subset of *trkB1* neurons (Fig 1A–1C and 1H, Table 1). Expression of *trkB1* coincides with *trpv1* while *trkC1* partially overlaps with *trpv1* (Fig 1D–1E, Table 1). This expression pattern is different than that described for the mouse DRG, where during early development *trpv1* is broadly coexpressed in TrkA neurons which specify nociceptors, but not in TrkB- or TrkC-expressing neurons which give rise mechanoreceptors and proprioceptors [14,15]. To confirm that in zebrafish *trkB1* and *trkC1* are indeed expressed in nociceptors, we compared their expression to *trpa1*. Expression of the zebrafish *trpa1b*:*GFP* transgene completely coincides with *trkC1* expression and is largely independent of *trkB1* expression. We also observed a small population of neurons that coexpressed a combination of *trkC1*, *trkB1*, *trpv1* and *trpa1b*:*GFP*. As *in situ* hybridization labeling has imperfect cellular resolution, we sought to confirm this small population expressing multiple markers by scatter labeling using CRSPR mediated insertion of the fluorescent reporter mRuby3 into the *trkB1* promoter region. Our *in situ* hybridization results predicted that ~19% (~6/~31) of *trkB1*-expressing TG neurons should coexpress *trpa1b*:*GFP* at 3dpf (Table 1). Similarly we found that 20% (15/74, n = 11 animals) of *trkB1*:*mRuby3*-expressing TG neurons co-expressed *trpa1b*:*GFP* (S1 Fig). Taken together these results demonstrate that *trkA*, *trkB1* or *trkC1* are largely expressed by all or the majority of nociceptive neurons as determined by *trpa1b*:*GFP* or *trpv1* expression during early larval development. This suggests that while the roles of neurotrophin receptors in somatosensory neuron survival and specification may be conserved, the distinct neuronal populations that these receptors represent are different between zebrafish and tetrapods.

**Table 1:** Trigeminal neuron counts.

Double positive cells	WT
*trkA+ trkB1+*	16.3±1.1 (3)
*trkA+ trkC1+*	3.7±0.4 (3)
*trkB1+ trkC1+*	5.5±0.8 (4)
*trkB1+ trpv1+*	30.7±1.5 (3)
*trkC1+ trpv1+*	11.3±1.5 (3)
*trkB1+ trpa1b*:*GFP+*	6.0±1.6 (4)
*trkC1+ trpa1b*:*GFP+*	13.0±1.9 (3)

Average number of double positive TG neurons for *trkA+ trkB1+*, *trkA+ trkC1+*, *trkB1+ trkC1+*, *trkB1+ trpa1b*:*GFP+*, and *trkC1+ trpa1b*:*GFP+* at 3dpf. Data represents mean±SEM (n).

### Zebrafish somatosensory neurons express *runx* and *cbfb*

**Fig 2:**
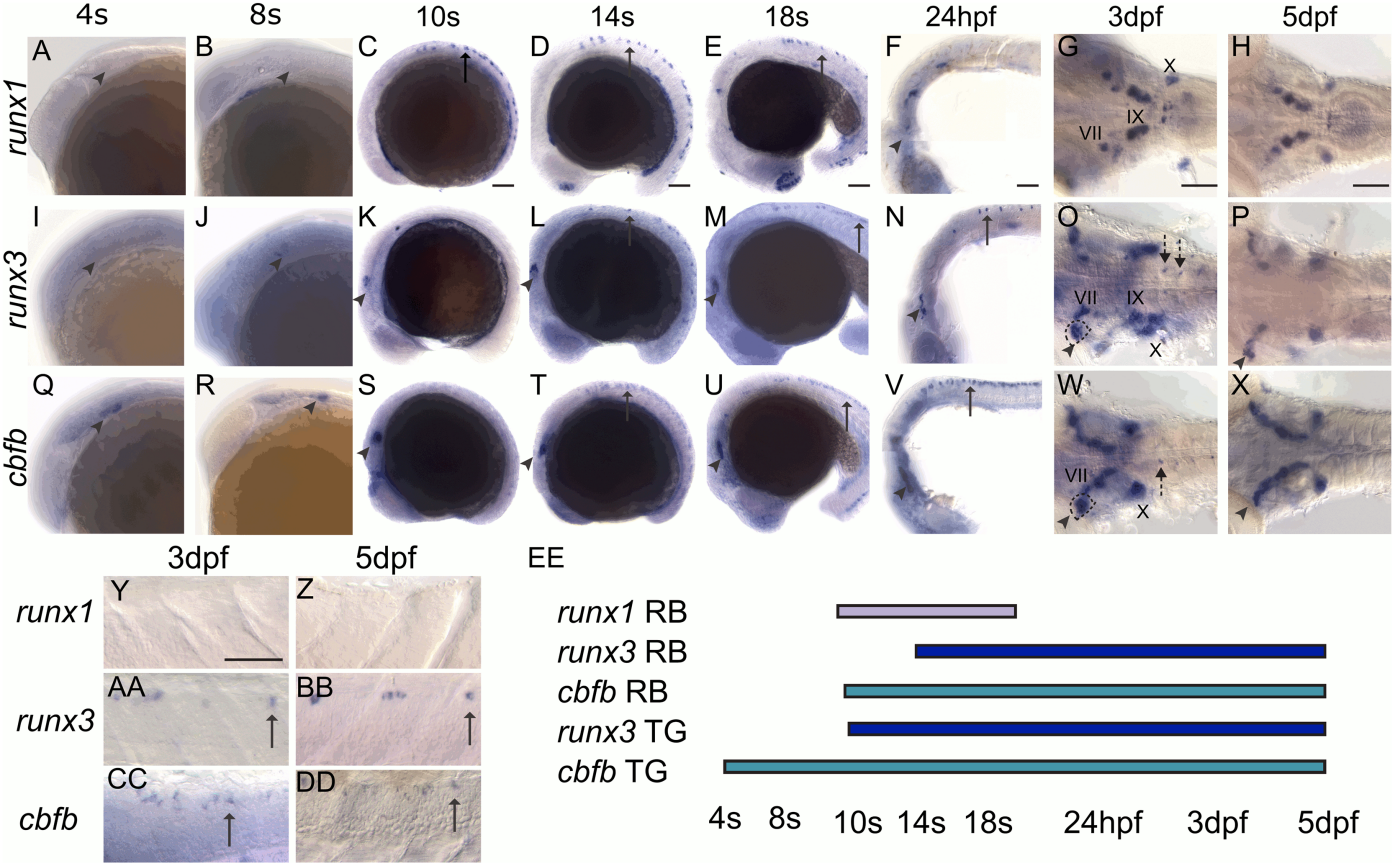
*runx1*, *runx3*, and *cbfb* are expressed in the zebrafish somatosensory system. A-DD, Colormetric *in situ* hybridization for *runx1* (A-H, Y-Z), *runx3* (I-P, AA-BB), and *cbfb* (Q-X, CC-DD) from 4s-5dpf. A-H, Y-Z, *runx1* is expressed in RBs (arrow) and can be detected in facial (VII), glossopharyngeal (IX), and vagal (X) cranial ganglion. I-P, AA-BB, *runx3* is expressed in RBs, in the DRG (dashed arrows), the TG (arrowhead) and in the VII, IX, X ganglion. I-P, AA-BB *cbfb* is expressed in RBs, in the DRG (dashed arrows), the TG (arrowhead) and in the VII, IX, X ganglion. EE, Rohon Beard and trigeminal expression timeline for *runx1*, *runx3*, and *cbfb*. Arrow indicates RBs; Arrowhead, TG; dashed arrow, DRG; VII, facial ganglion; IX, glossopharyngeal ganglion; X, vagal ganglion. Scale bar: 100 μm.

To determine if transcriptional regulators of Trk expression and somatosensory neuron diversification are conserved between fish and tetrapods, we investigated the role of zebrafish Runx TFs and their cofactor Cbfβ in neuronal fate specification. We first characterized the expression of *runx1*, *runx3*, and *cbfb* using colormetric RNA *in situ* hybridization across multiple timepoints. *runx1* is only expressed in Rohon-Beard (RB) sensory neurons, which innervate the body, while *runx3* and *cbfb* are expressed in all three somatosensory neuron populations: RB, DRG, and TG neurons (Fig 2). All three genes are also expressed in other cranial ganglion, such as the facial (VII), glossopharyngeal (IX), and vagal (X) ganglia (Fig 2). In RB neurons, *runx1* is transiently expressed with expression observed at the 4 somite (s) stage, but absent by 24hpf (hours post fertilization) (Fig 2A–2D, 2S, 2T and 2Y). In contrast, *runx3* and *cbfb* RB expression begins later at 14s and continues to at least 5dpf (days post fertilization) (Fig 2G–2R and 2U–2Y). In the TG, *cbfb* expression was detected as early as 4s, while definitive *runx3* expression was observed at 10s. Both continued to be expressed till at least 5dpf, while we found no evidence of *runx1*-expression even at the earliest timepoints measured (Fig 2A-X and 2EE). These data suggest that Runx TFs are in a position to regulate zebrafish somatosensory neuron diversification. However the temporal expression of *runx1* in RB neurons and the lack of expression of *runx1* in the TG suggest that individual Runx TFs may have specialized roles in tetrapods that are not required in larval teleost fish.

### *runx1* and *runx3* act in the same pathway and are regulated differently from *cbfb*

**Fig 3:**
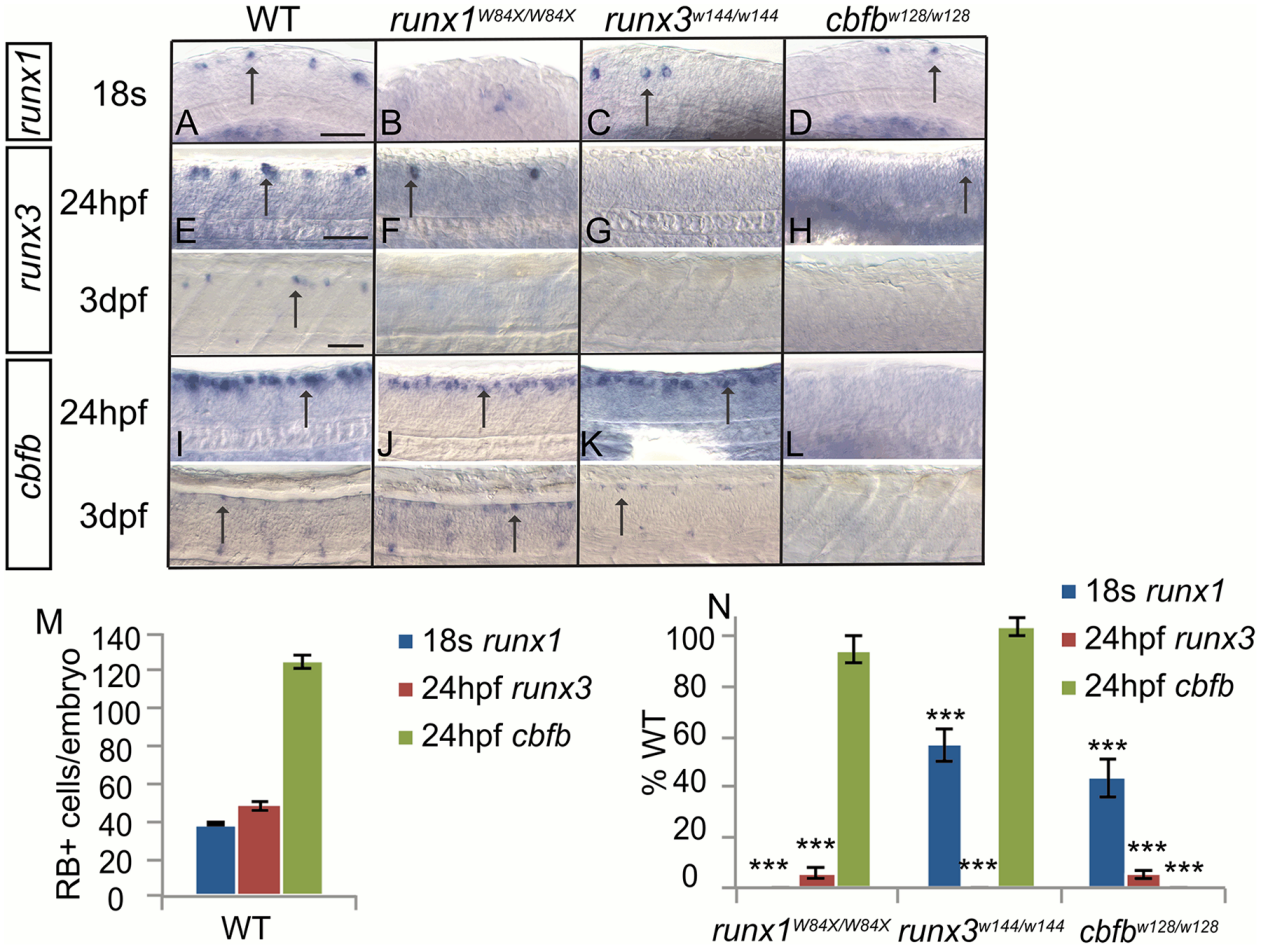
Runx/Cbfβ signaling is required to maintain *runx* expression in Rohon Beard neurons. A-L, Colormetric *in situ* hybridization for *runx1* (A-D) at 18s, *runx3* (E-H) at 24hpf and 3dpf, and *cbfb* (I-L) at 24hpf and 3dpf in the runx*1*, *runx3*, and *cbfb* mutants focusing on the RBs. M-N, Quantification of marker gene expression as total number of RB neurons/embryo and as % WT at 18s or 24hpf. Arrow, RBs; Scale bar: 100 μm. Embryos per condition (n = 3–14). ***p<0.001. All error bars represent S.E.M.

**Fig 4:**
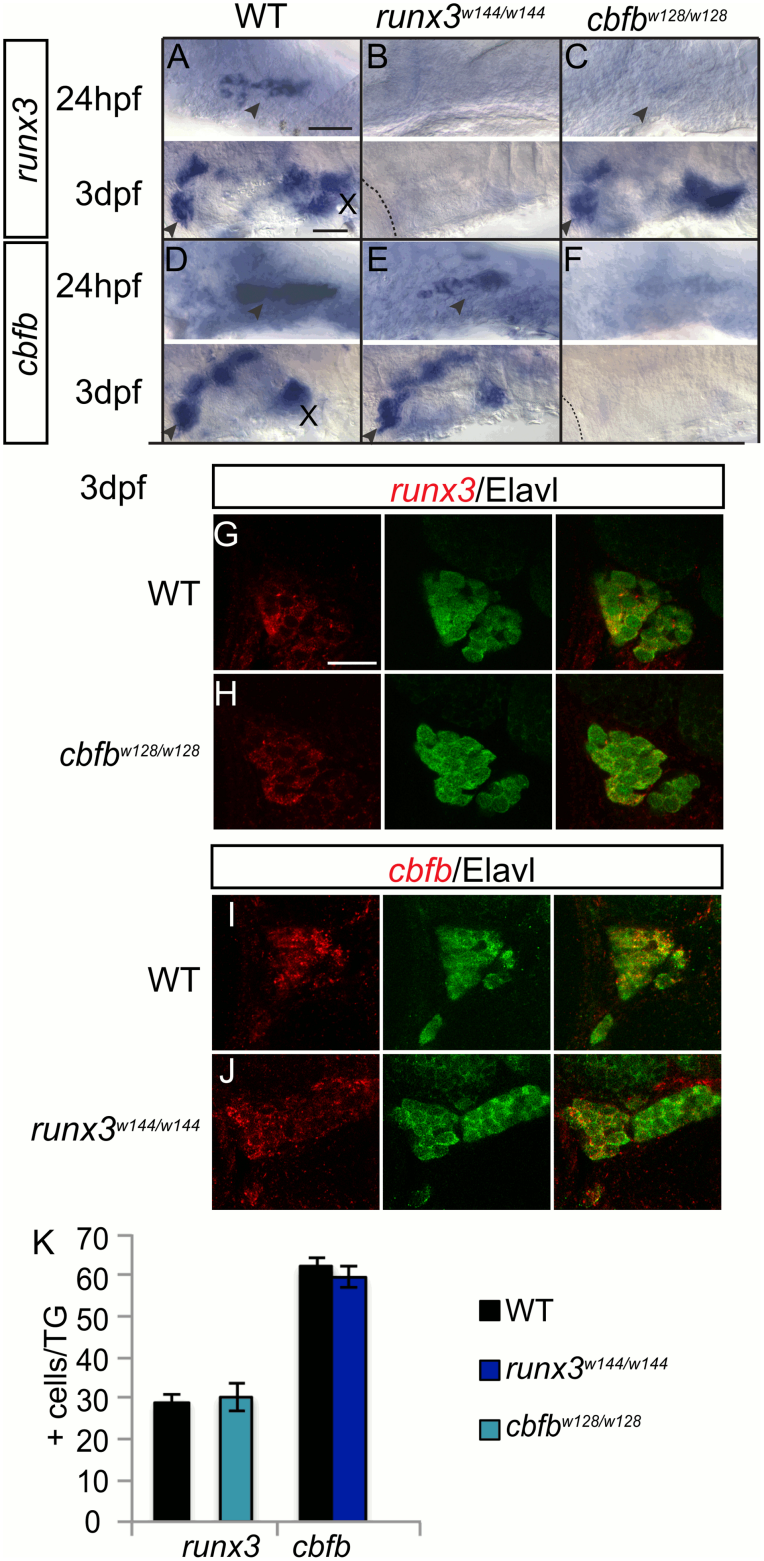
Cbfβ expression does not affect *runx* expression in the TG. A-F, Colormetric *in situ* hybridization for *runx3* (A-C) and *cbfb* (D-F) in the *runx3*^*w144/w144*^ and *cbfb*^*w128/w128*^ mutants focusing on the TG at 24hpf and 3dpf. G-J, Antibody staining of Elavl (green) to label the whole trigeminal in conjunction fluorescent *in situ* hybridization for *runx3* (G-H) and *cbfb* (I-J) in *runx3*^*w144/w144*^ and *cbfb*^*w128/w128*^ mutants at 3dpf. K, Quantification of marker gene expression as total number of TG neurons/ganglion at 3dpf. Dashed line outlines the eye; Arrowhead, TG; X, vagal ganglion. Scale bar: A-F 100 μm, G-J 20μm. Embryos per condition (n = 3–7). All error bars represent S.E.M.

To examine the effect of impaired Runx function in somatosensory neuron development, we obtained a *runx1*^*W84X*^ truncation mutant and generated mutations in *runx3* and *cbfb* using CRISPRs and TALENs, respectively [16–18]. We created two nonsense mutations, *runx3*^*w144*^ (1bp del) and *cbfb*^*w128*^ (4bp del); all three mutations predict an early truncation (S2 Fig). I*n situ* hybridization verified the lack of the gene expression in homozygous mutants, most likely caused by nonsense-mediated decay of the RNA transcript (Figs 3B, 3G and 3L and 4B and 4F). All mutants were viable and showed no obvious deformities during the time course of the experiments conducted. However all mutations induced lethality 8-10dpf. In the RBs of wild-type (WT) fish, we found that *cbfb* was expressed in a large proportion (~76%) of RB cells, while *runx3* and *runx1* expression was more tightly restricted (Fig 3A, 3E, 3I and 3M; Table 2). Mutations in *runx3* or *cbfb* resulted in a reduction to about half of the *runx1*+ cells found in WT at 18s stage (Fig 3A, 3C, 3D and 3N; Table 2). Similarly, mutations of *runx1* or *cbfb* resulted in an almost complete loss of *runx3* expression in RBs at 24hpf and complete absence by 3dpf (Fig 3F, 3H and 3N; Table 2). As a result in relation to RB neurons the *runx1* mutants could be considered a de facto double null mutant for *runx1* and *runx3*. By contrast there was no change in *cbfb* expression in RB cells at 24hpf or 3dpf in *cbfb*, runx1 or *runx3* mutants (Fig 3J, 3K and 3N; Table 2). Animals heterozygous for mutations in both *runx1* and *runx3* had significantly fewer *runx1*- and *runx3*-expressing RB neurons suggesting that in this neuronal population, Runx1 and Runx3 are functioning in the same pathway (S3 Fig). Animals heterozygous for mutations in *cbfb* and either *runx1* or *runx3* however had no effect on *runx1*, *runx3* or *cbfb* expression (S3 Fig). We can therefore conclude that in the RB population, Runx and Cbfβ help maintain *runx1* and *runx3* expression and that a cumulative loss of Runx1 and/or Runx3 inhibits the ability of Runx proteins to facilitate *runx1* and *runx3* expression. Loss of either *runx* gene, however, does not impact *cbfb* expression, indicating that *cbfb* expression is regulated by a Runx independent mechanism.

**Table 2:** Rohon Beard neuron counts.

Counts	WT	*runx1*^*W84X/W84X*^	*runx3*^*w144/w144*^	*cbfb*^*w128/w128*^
18s *runx1*	38.8±1.1 (14)	0±0 (6)	22.0±2.4 (7)	17.0±3.0 (3)
24hpf *runx3*	48.2±2.9 (5)	2.9±1.0 (3)	0±0 (7)	2.6±0.9 (4)
24hpf *cbfb*	124.8±3.4 (10)	118.3±6.8 (4)	129.8±5.1 (8)	0±0 (5)

Average number of *runx1*, *runx3* or *cbfb* positive Rohon Beard neurons at 18s or 24hpf. Data represents mean±SEM (n).

We found different regulatory patterns in the TG, where *runx3*, but not *runx1* is expressed. TG somatosensory neurons were identified based on their location and their expression of Elavl, a pan-neuronal marker [19]. In 3dpf wild-type fish, *cbfb* was expressed in all TG neurons while *runx3*+ neurons comprised of less than half of the TG population (Fig 4G, 4I and 4K, Table 3). Although at 24hpf, there is a transient reduction in *runx3* expression in the *cbfb* mutant, by 3dpf the number of *runx3*-expressing neurons is no different than WT (Fig 4A–4C, 4G, 4H and 4K; Table 3). *cbfb* expression also appeared unchanged in the *runx3* mutant at 24hpf and 3dpf in the TG and the total number of *cbfb*+ cells at 3dpf was unchanged (~96% of WT) (Fig 4D–F, 4I and 4J; Table 3). Consistent with our findings that *runx1* is not expressed in the TG, we saw no gross effect on *runx3* expression in runx1 mutants at 3 dpf (S4A and S4B Fig). These data suggest that *cbfb* is not required for the survival of *runx3*-expressing TG neurons and vice versa. These results are similar to what has been reported in mouse DRG, where *runx* expression is independent of Runx activity [2].

**Table 3:** Trigeminal ganglion neuron counts.

neurons/ganglion	WT	*runx3*^*w144/w144*^	*cbfb*^*w128/w128*^
*runx3*	29.3±1.8 (7)		30.3±3.3 (3)
*cbfb*	62.25±1.9 (4)	59.6±2.5 (5)	

Average number of *runx3* or *cbfb* positive TG neurons as identified by Elavl staining at 3dpf in the *runx3*^*w144/w144*^ and *cbfb*^*w128/w128*^ mutants. Data represents mean±SEM (n).

### Loss of Runx function does not affect the total number of neurons or apoptosis in the TG or RBs

In some contexts, loss of mammalian Runx function results in sensory neuron death [4,20,21]. We tested whether mutation of zebrafish *runx* or *cbfb* genes affected neuronal number in the TG and the RBs. None of the mutants showed any change in the number of RB neurons at 24hpf (Table 4; S5A–S5D and S5H Fig). There was also no change in the number of cells in the TG at 24 hpf or 3dpf (Table 4; S5E–S5G and S5I Fig). We next investigated if loss of Runx activity affected caspase-3 dependent apoptosis. Wildtype, *runx1*, *runx3* and *cbfb* mutants were stained for activated caspase-3 at 24, 36, 48 and 72 hpf. We observed little activated caspase-3+ apoptotic cells in the TG at any of the timepoints and no difference among the genotypes (Table 5; S6 Fig). Within the time frame observed, these data demonstrate that loss of Runx signaling in zebrafish does not lead to an increase in neuronal cell death or changes in neuron number, though we cannot rule out changes at later timepoints.

**Table 4:** Elavl neuron counts.

	WT	*runx1*^*W84X/W84X*^	*runx3*^*w144/w144*^	*cbfb*^*w128/w128*^
24hpf RB+ cells	165.1±4.1 (5)	162.3±8.2 (3)	159.0±5.6(3)	159.5±4.0 (4)
24hpf TG+ cells	26.0±3.2 (3)		29.3±2.7 (3)	24.5±2.0 (4)
3dpf TG+ cells	63.0±0.9 (26)		64.1±3.4 (6)	62±5.0 (3)

Average number of Elavl+ 24hpf RB, 24hpf TG, and 3dpf TG cells in the *runx1*^*W84X/W84X*^, *runx3*^*w144/w144*^, and *cbfb*^*w128/w128*^ mutants. Data represents mean±SEM (n).

**Table 5:** Caspase 3 positive neurons counts.

Age	WT	*runx1*^*W84X/W84X*^	*runx3*^*w144/w144*^	*cbfb*^*w128/w128*^
1dpf	1.40± .46 (5)	0.67± .54 (3)	1.25± .21 (4)	1.75± .37 (4)
1.5dpf	0.85± .32 (7)	1 ± 0.49 (5)	1 ± 0.40 (6)	0.66 ± 0.30(6)
2dpf	0.20± .18 (5)	0.5 ± 0.25 (4)	0.00± 0.0 (5)	0.40± .22 (5)
3dpf	0.60± .36 (5)	0.2± .22 (4)	0.80± .33 (5)	0.75± .21 (5)

Average number of Caspase3 positive TG neurons at 24, 36, 48, 72 hours post fertilization in the *runx1*^*W84X/W84X*^, *runx3*^*w144/w144*^, and *cbfb*^*w128/w128*^ mutants. Data represents mean±SEM (n).

### *runx* and *cbfb* are required for proper Trk receptor expression

**Fig 5:**
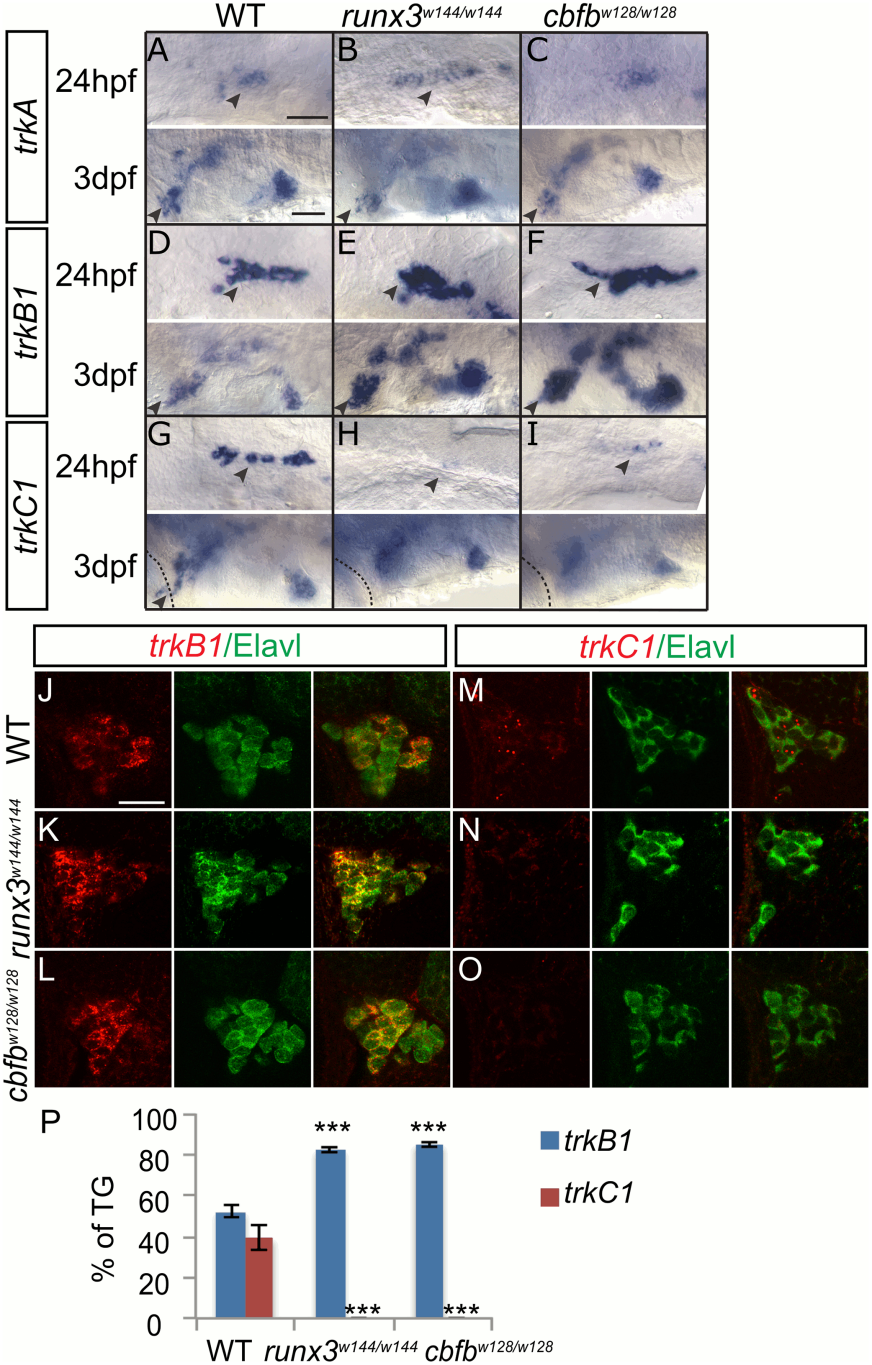
Loss of *runx* or *cbfb* expression affects *trk* receptor expression in the TG. A-I, Colormetric *in situ* hybridization for *trkA* (A-C), *trkB1* (D-F), and *trkC1* (G-I) in the *runx3*^*w144/w144*^ and *cbfb*^*w128/w128*^ mutants focusing on the TG at 24hpf and 3dpf. J-O, Antibody staining of Elavl (green) in conjunction with fluorescent *in situ* hybridization for *trkB1* (J-L) and *trkC1* (M-O) in the *runx3*^*w144/w144*^ and *cbfb*^*w128/w128*^ mutants at 3dpf. P, Quantification of marker gene expression as total number of TG neurons/ganglion and as % TG at 3dpf. Arrowhead, TG; X, vagal ganglion. Scale bar: A-I 100 μm, J-O 20μm. Embryos per condition (n = 3–5). ***p<0.001. All error bars represent S.E.M.

Although there is some discrepancy between different studies, it is clear that Runx regulates Trk receptor expression in mammals. Conditionally knocking out Runx1 in the DRG neurons of mice results in the expansion of TrkA-expressing neurons [7]. In Runx3 knockouts, some studies found an increase in TrkB-expressing neurons in the DRG and TG although other studies reported no change in the DRG [4,6]. Runx3 knockouts also showed either a loss of TrkC+ neurons in the DRG or a decrease in TrkC expression in the DRG and TG [4,6,22]. We performed *in situ* hybridization for *trk* receptors in each zebrafish mutant to determine whether Runx could play similar roles in regulating *trk* receptor expression. Analysis of the TG is simplified as only *runx3* but not *runx1* is expressed. *trkA* expression was unchanged in the TG at 24hpf and 3dpf in the *runx3* or *cbfb* mutant (Fig 5A–5C). *trkB1* expression increased at 24hpf and 3dpf in the *runx3* and *cbfb* mutant while *trkC1* expression was reduced in the TG at 24hpf and absent at 3dpf (Fig 5D–5I). To quantify these changes, we performed fluorescent *in situ* hybridization and counterstained for Elavl. In WT, we found that ~55% of the TG expressed *trkB*1 and ~42% of the TG expressed *trkC1* (Fig 5P). In both *runx3* and *cbfb* mutants we observed a significant increase in the number of *trkB1*+ neurons to ~89% of total and a complete loss in the number of *trkC1*+ neurons (Fig 5J–5P; Table 6). Surprisingly, there was no apparent change in *trkC1* expression in other cranial ganglia, such as the facial and vagal ganglia that also express *runx* and *cbfb*, suggesting that neurotrophin receptor expression is regulated by an alternative mechanism in these structures.

**Table 6:** Trigeminal ganglion neuron counts.

	WT	*runx3*^*w144/w144*^	*cbfb*^*w128/w128*^
*trkB1*	35.0±0.5 (4)	55.4±2.8(5)	57.0±5.8 (3)
*trkC1*	26.4±4.4 (3)	0.0±0.0 (3)	0.0±0.0 (3)

Average number of *trkB1* or *trkC1* positive TG neurons as identified by Elavl at 3dpf in the *runx3*^*w144/w144*^ and *cbfb*^*w128/w128*^ mutants. Data represents mean±SEM (n).

**Fig 6:**
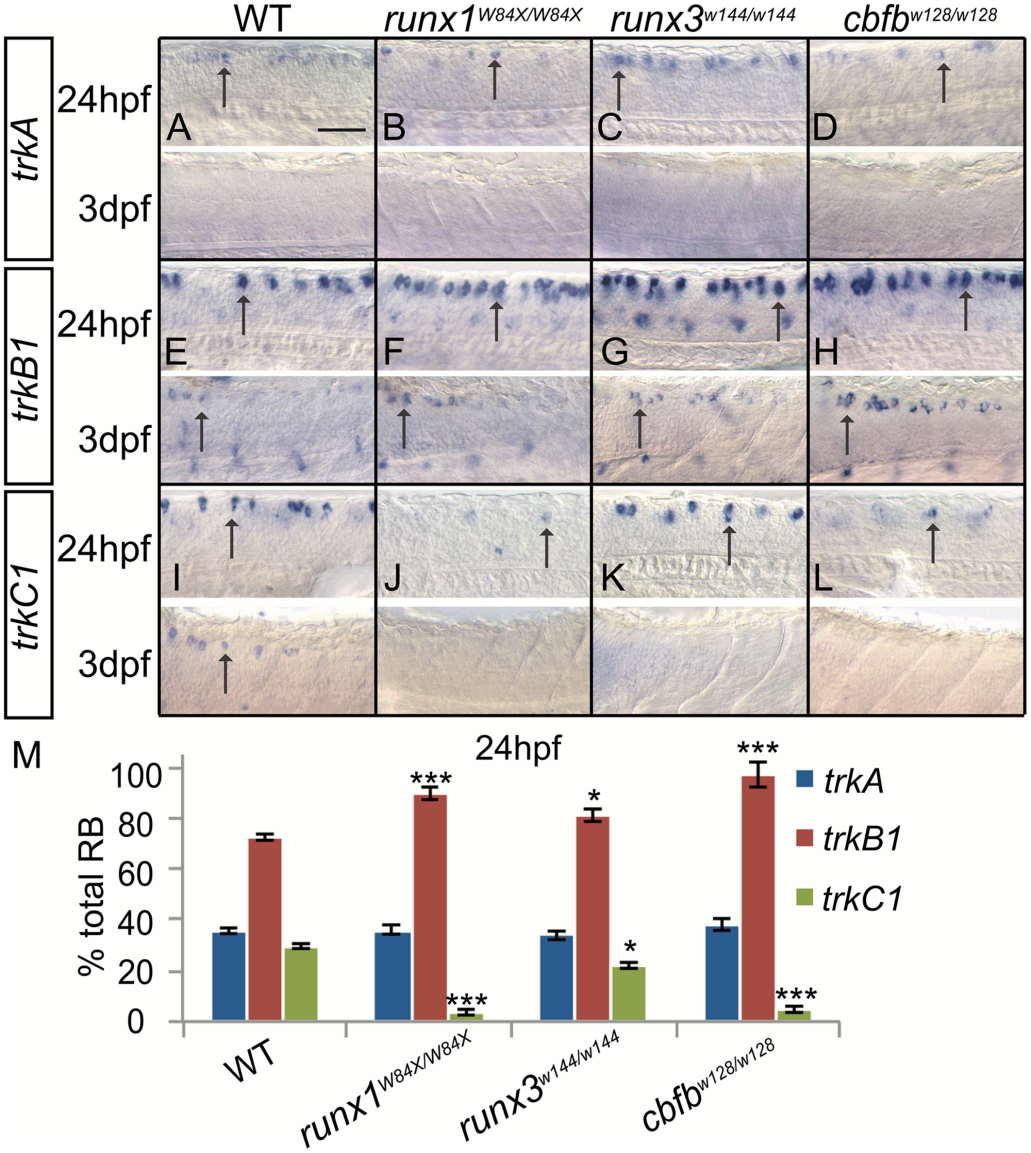
Loss of *runx* or *cbfb* expression affects *trk* receptor expression in the RBs. A-M, Colormetric *in situ* hybridization for *trkA* (A-D), *trkB1* (E-H), and *trkC1* (I-L) in *runx1*^*W84X/W84X*^, *runx3*^*w144/w144*^ and *cbfb*^*w128/w128*^ mutants focusing on the RBs at 24hpf and 3dpf. M, Quantification of marker gene expression as % total RBs at 24hpf. Arrow, RBs. Scale bar: 100 μm. Embryos per condition (n = 3–12). *p<0.05, ***p<0.001. All error bars represent S.E.M.

*trkA* expression was also unchanged in the RBs at 24hpf in the runx*1*, *runx3* and *cbfb* mutants (Fig 6A–6D; Table 7); we were unable to detect *trkA* expression in RBs in 3dpf larvae. The number of *trkB1*+ RB cells was increased at 24hpf and 3dpf in all three mutants while *trkC1* expression was reduced at 24hpf and absent at 3dpf (Fig 6E–6N; Table 7). At 24hpf, *trkB1*+ neurons were about 70% and *trkC1*+ neurons were about 30% of the total RB neurons (Fig 6M; Table 7). In the runx1 and *cbfb* mutant, there was a significant increase of *trkB1* expression, where almost all of the RB neurons expressed *trkB1*, and a corresponding decrease such that almost no RB neurons expressed *trkC1* (Fig 6M; Table 7). The *runx3* mutant showed a less dramatic, but still significant increase in *trkB1*+ neurons and decrease *trkC1*+ neurons at 24hpf, potentially due to compensation by early *runx1*+ expression; however by 3dpf, the distribution of expression in the *runx3* mutant was indistinguishable from the other mutants (Fig 6M; Table 7). Furthermore *runx1*/*runx3* heterozygous animals showed a significant increase in *trkB1*+ neurons and decrease *trkC1*+ neurons at 24hpf (S3 Fig).

**Table 7:** Rohon Beard neuron counts.

	WT	*runx1*^*W84X/W84X*^	*runx3*^*w144/w144*^	*cbfb*^*w128/w128*^
*trkA*	58.7±1.6 (12)	59.33±3.2 (3)	56.0±2.8 (3)	62.6±3.5 (5)
*trkB1*	119.2±2.4 (6)	148.0±3.9 (5)	133.3±4.3 (3)	160.0±7.9 (3)
*trkC1*	48.7±1.3 (8)	5.8±1.5 (4)	36.0±1.5 (7)	7.8±2.2 (4)

Average number of *trkA*, *trkB1* or *trkC1* positive RB neurons at 24hpf in the *runx1*^*W84X/W84X*^, *runx3*^*w144/w144*^, and *cbfb*^*w128/w128*^ mutants. Data represents mean±SEM (n).

The RB and TG data are very similar and support a role for *runx* and *cbfb* in suppressing *trkB1* expression and promoting *trkC1* expression. Since the increase in *trkB1*+ neurons is mirrored with a decrease in *trkC1*+ neurons and there is no change in neuronal number or apoptosis, we suggest that Runx/Cbfβ activity suppresses *trkB1* expression and promotes *trkC1* expression within the same subset of neurons. While apparently having no role in regulating *trkA* expression, a role Runx1 plays in mammals, the ability of Runx3 to regulate *trkB1* and *trkC1* expression is conserved between larval fish and terrestrial vertebrates even though these receptors label different neuronal subtypes in these species.

### Loss of *runx3* leads to the loss of some nociceptive receptors

**Fig 7:**
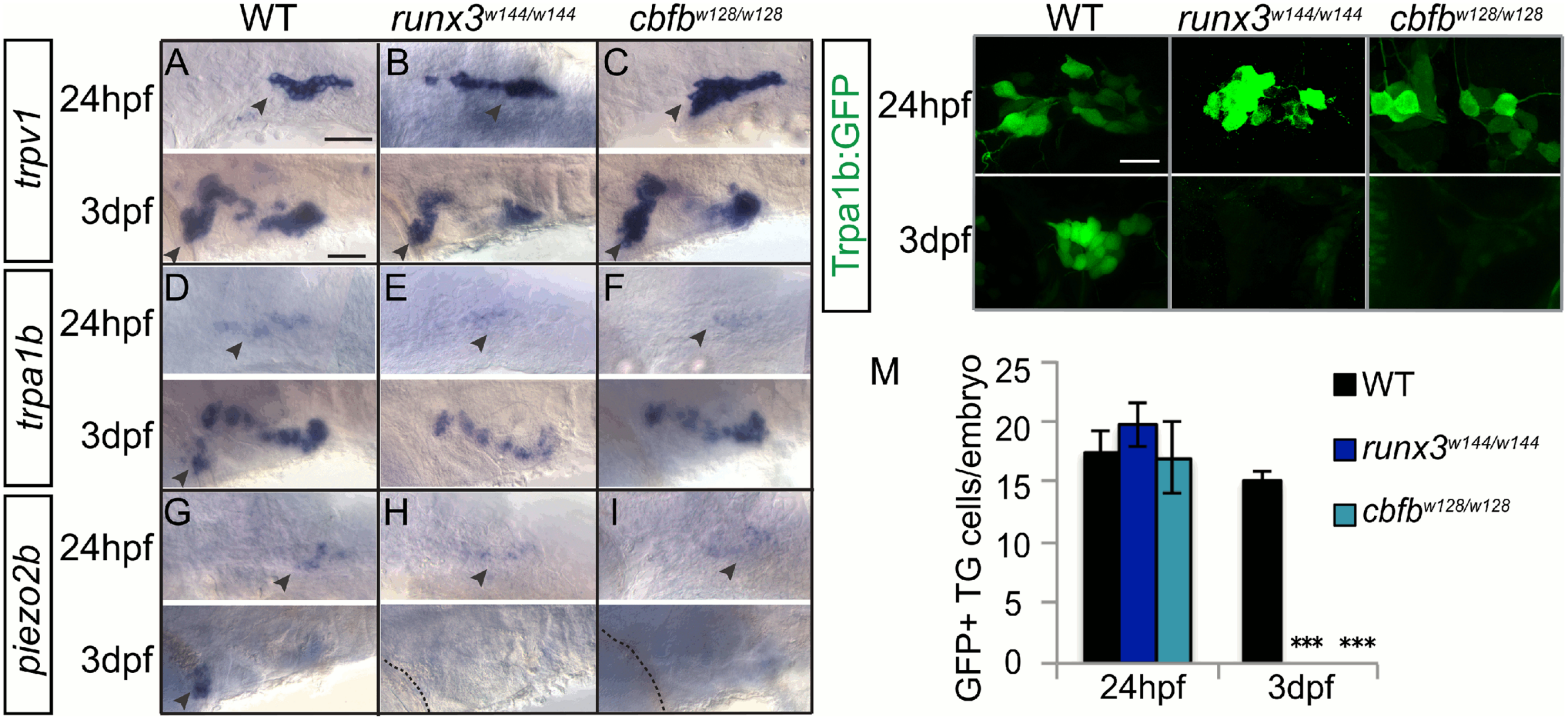
Loss of *runx* or *cbfb* expression affects ion channel expression in the TG. **A-I**, Colormetric *in situ* hybridization for *trpv1* **(A-C)**, *trpa1b* **(D-F)**, and *piezo2b* **(G-I)** in the *runx3*^*w144/w144*^ and *cbfb*^*w128/w128*^ mutants focusing on the TG at 24hpf and 3dpf. **J-L**, Antibody staining of GFP (green) in *runx3*^*w144/w144*^; *trpa1b*:*GFP* and *cbfb*^*w128/w128*^; *trpa1b*:*GFP* mutants. **M**, Quantification of GFP+ cells/ganglion in the TG at 24hpf and 3dpf. Dashed line outlines the eye; Arrowhead, TG. Scale bar: A-I 100 μm, J-L 20μm. Embryos per condition (n = 3–7). ***p<0.001. All error bars represent S.E.M.

In mammals, loss of Runx1 leads to decreased or complete loss of expression of sensory ion channels including the noxious heat receptor TRPV1, the noxious chemosensor TRPA1, the light touch receptor Piezo2 and nociceptor specific voltage gated sodium channel NaV1.9 in the DRG, with corresponding somatosensory behavioral deficits [7,8]. In the TG, we examined expression of the zebrafish orthologs *trpv1*, *trpa1b*, *piezo2b*, and *scn1α* (NaV1.7 ortholog) in *runx3* and *cbfb* mutants. The phenotypes of the *runx3* and *cbfb* mutants were identical. *trpv1* and *scn1a* expression appeared normal at 24hpf and 3dpf (Fig 7A–7C). By contrast, *trpa1b* expression by *in situ* and by GFP expression in the *trpa1b*:*GFP* transgenic reporter line was present at 24hpf, but absent at 3dpf (Fig 7D–7F and 7J–7M; Table 8). Loss of *trpa1b* expression was highly specific to the TG as expression was normal in other cranial ganglia, again suggesting that the effects of Runx/Cbfβ signaling are specific to somatosensory neurons. *piezo2b* expression was also present at 24hpf, but was completely gone from the TG by 3dpf (Fig 7G–7I). Consistent with Runx1 not having a role in TG neuronal specification, we observed no change in the number of GFP expressing neurons in runx1 mutants with the *trpa1b*:*GFP* transgenic reporter (Table 8; S4C and S4D Fig)

**Table 8:** Trigeminal ganglion neuron counts.

	WT	*runx1*^*W84X*^	*runx3*^*w144/w144*^	*cbfb*^*w128/w128*^
24hpf *trpa1b*:*GFP*+	17.5±1.8 (3)		19.7±1.7(3)	17.0±3.1 (3)
3dpf *trpa1b*:*GFP*+	15.1±0.9 (7)	14.2±0.7 (13)	0.0±0.0 (5)	0.0±0.0 (5)
24hpf *Isl1SS*:*Kaede*+	17.2±1.3 (5)			17.0±3.1 (3)
3dpf *Isl1SS*:*Kaede*+	11.1±1.2 (7)			11.0±1.6 (3)
24hpf *p2x3b*:*eGFP*+	16.2±2.3 (6)			15.6±2.1 (3)
3dpf *p2x3b*:*eGFP*+	24.5±3.5 (5)			26.0±2.6 (3)

Average number of GFP or Kaede positive TG neurons at 3dpf in the *runx3*^*w144/w144*^ and *cbfb*^*w128/w128*^ mutants in the *trpa1b*:*GFP*, *Isl1SS*:*Kaede* and *p2x3b*:*eGFP* transgenic fish. Data represents mean±SEM (n).

**Fig 8:**
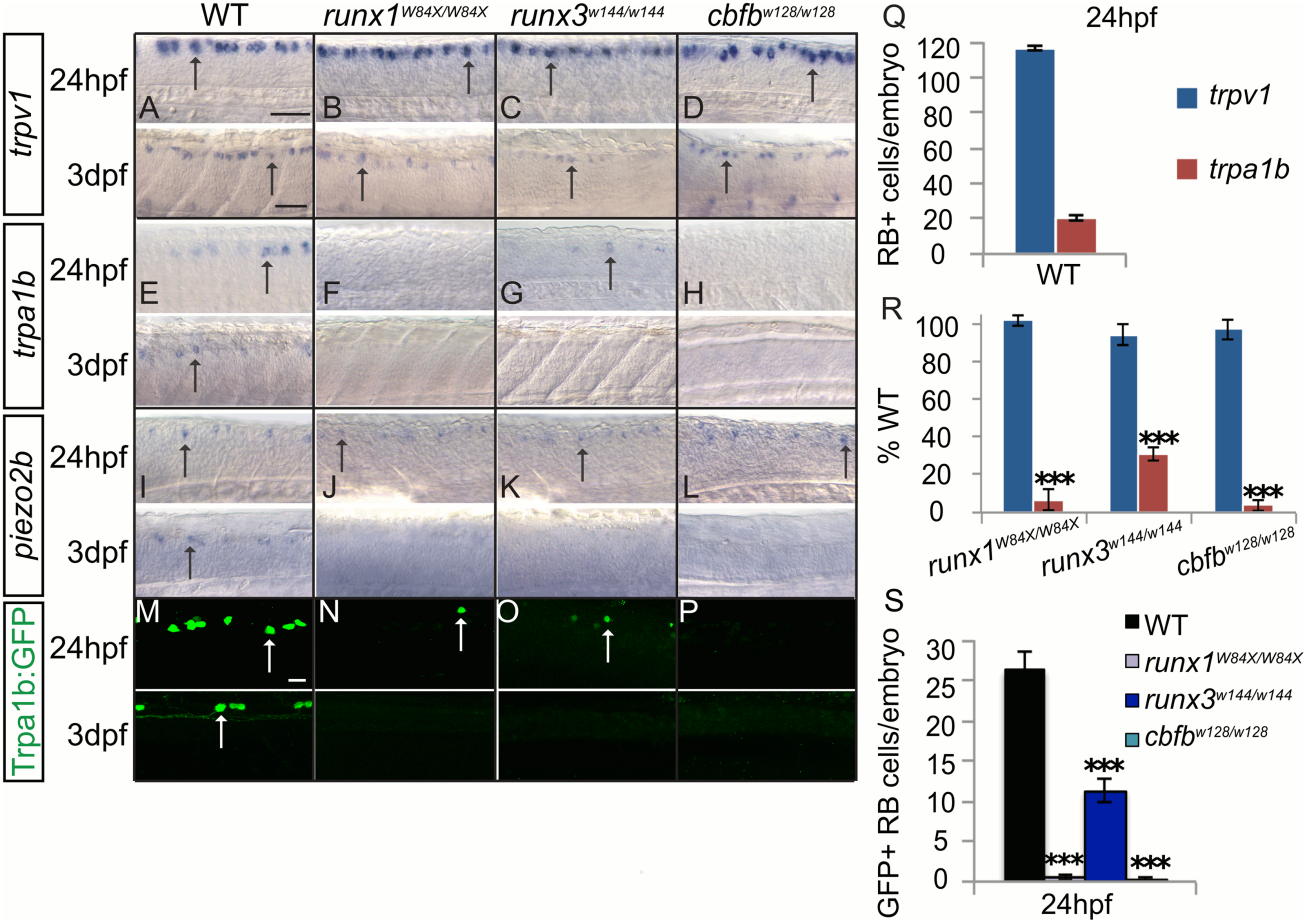
Loss of *runx* or *cbfb* expression affects ion channel expression in the RBs. **A-L**, Colormetric *in situ* hybridization for *trpv1* **(A-D)**, *trpa1b* **(E-H)**, and *piezo2b* **(I-L)** in the runx*1*^*W84X/W84X*^, *runx3*^*w144/w144*^ and *cbfb*^*w128/w128*^ mutants focusing on the RBs at 24hpf and 3dpf. **M-P**, Antibody staining of GFP (green) in *runx3*^*w144/w144*^; *trpa1b*:*GFP* and *cbfb*^*w128/w128*^; *trpa1b*:*GFP* mutants. **Q-R**, Quantification of marker gene expression as total number of RB neurons/embryo and as % WT at 24hpf. **S**, Quantification of GFP+ cells in the RBs at 24hpf. Dashed line outlines the eye; Arrow, RBs; Arrowhead, TG; X, vagal ganglion. Scale bar: A-L 100 μm, J-L, M-N 20μm. Embryos per condition (n = 3–12). ***p<0.001. All error bars represent S.E.M.

The phenotype in the RB neurons mirrored the TG phenotype. The expression of *trpv1* and *scn1a* was unchanged at 24hpf and 3dpf (Fig 8A–8D and 8Q–8R; Table 9; S7P–S7T Fig). The expression of *trpa1b* as measured by *in situ* and GFP expression in the *trpa1b*:*GFP* transgenic was significantly reduced at 24hpf and absent at 3dpf in all three mutant lines (Fig 8E–8H and 8M–8P; Table 9). *piezo2b* expression was normal at 24hpf, but was absent from RB neurons at 3dpf (Fig 8I–8L). Similar to *trkC1* expression, Runx/Cbfβ is required for maintenance of *trpa1* and *piezo2b* expression but not for initiation. These data suggests that in larval zebrafish only some aspects of mammalian Runx function in regulating the expression of different sensory ion channels and other nociceptor markers were present. However, these functions are dependent on Runx3 and not Runx1 signaling.

**Table 9:** Rohon Beard neuron counts.

	WT	*runx1*^*W84X/W84X*^	*runx3*^*w144/w144*^	*cbfb*^*w128/w128*^
*trpv1*	116.3±1.0 (12)	118.7±3.5 (6)	110.0±6.7 (3)	113.0±6.2 (4)
*trpa1b*	20.1±1.2 (12)	1.3±1.1 (3)	6.3±0.7 (4)	0.8±0.6 (4)
*trpa1b*:*GFP*	26.2±2.4 (6)	0.5±0.3 (4)	11.3±1.5 (3)	0.3±0.3 (4)
*scn1α*	128.3±3.4 (11)	121.0±3.7 (3)	120.7±4.3 (3)	135.7±8.2 (3)

Average number of *trpv1*, *trpa1b*, *trpa1b*:*GFP*, and *scn1α* positive RB neurons at 24hpf in the *runx1*^*W84X/W84X*^, *runx3*^*w144/w144*^, and *cbfb*^*w128/w128*^ mutants. Data represents mean±SEM (n).

We also asked whether loss of zebrafish Runx function changed expression of markers that define nociceptive neuron subtypes in the mouse, such as CGRP, Ret and the ATP receptor P2X3 [7,8]. We examined mRNA expression of *cgrp* and *ret*, as well as GFP expression in transgenic reporter lines *Isl1SS*:*Kaede*, which labels a subpopulation of peptidergic nociceptors, and *P2x3b*:*eGFP* in the *runx3* and *cbfb* mutants [3,23]. In the TG, we saw no change in any of these markers at any of the timepoints we examined (Table 8; S7A–S7F and S7J–S7O Fig). Unfortunately we were unable to document clear and consistent expression of *cgrp* and *ret* in RB neurons. We can conclude however that in zebrafish TG, Runx3/Cbfβ signaling is not involved in regulating the expression of markers of peptidergic and nonpeptidergic nociceptors. These functions were likely acquired in terrestrial vertebrates alongside Runx1 specialization in specification of nociceptor cell fate.

**Fig 9:**
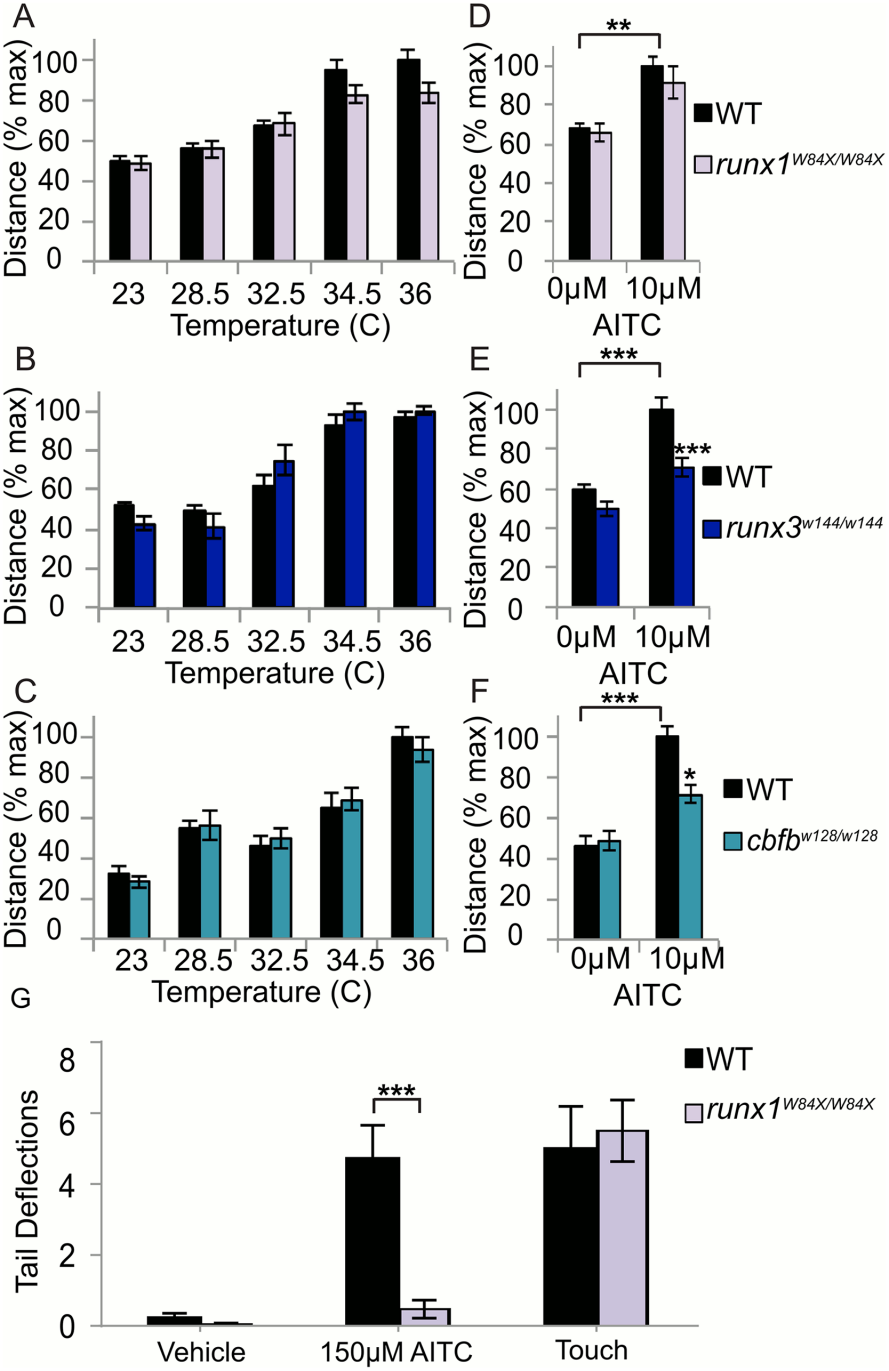
Loss of *runx* or *cbfb* expression affects locomotor responses of zebrafish to AITC, but not heat. **A-C**, Heat behavior in the *runx1*^*W84X/W84X*^ (**A**), *runx3*^*w144/w144*^ (**B**), and *cbfb*^*w128/w128*^ (**C**) mutants. **D-F**, AITC behavior in the *runx1* (**D**), *runx3* (**E**), and *cbfb* (**F**) mutants **(G)** AITC and touch tail deflection behavior of 3dpf *runx1* larvae. Embryos per condition (n = 10–25). *p<0.05, **p<0.01, ***p<0.001. All error bars represent S.E.M.

To determine whether changes in *trpa1b* ion channel expression had functional consequences, we used a locomotor assay at 5 dpf to test whether Runx mutants showed defects in their responses to heat and AITC [13]. *runx1*, *runx3* and *cbfb* mutants showed no behavioral change in response to heat compared to WT fish (Fig 9A–9C), reflecting our earlier findings showing no change in *trpv1* ion channel expression. In contrast, the locomotor responses of 5dpf *runx3* and *cbfb* mutants to AITC were abolished, which reflects the loss of *trpa1b* expression specifically in the TG and RB of these mutants (Fig 9E and 9F). However, runx1 mutants, which have normal *trpa1b* expression in the TG but a loss of *trpa1b* expression in RBs, responded normally to AITC (Fig 9D). To determine if the loss of Trpa1b in RB neurons could affect localized behavioral responses in *runx1* mutants, we performed a tail-deflection assay in which AITC was puffed onto the tails of head immobilized 5dpf larvae and tail flick responses were monitored. Tail-deflection to AITC was almost completely abolished in *runx1* mutants compared to WT controls while responses to a mechanical stimulus were unaffected (Fig 9G; S1–S6 Videos). This result indicates that while *trpa1b* expression in RB neurons is required for localized sensitivity to AITC in the body/tail, expression in the TG is sufficient to evoke WT levels of locomotion in response to bath applied AITC.

### Runx can rescue loss of Cbfβ

**Fig 10:**
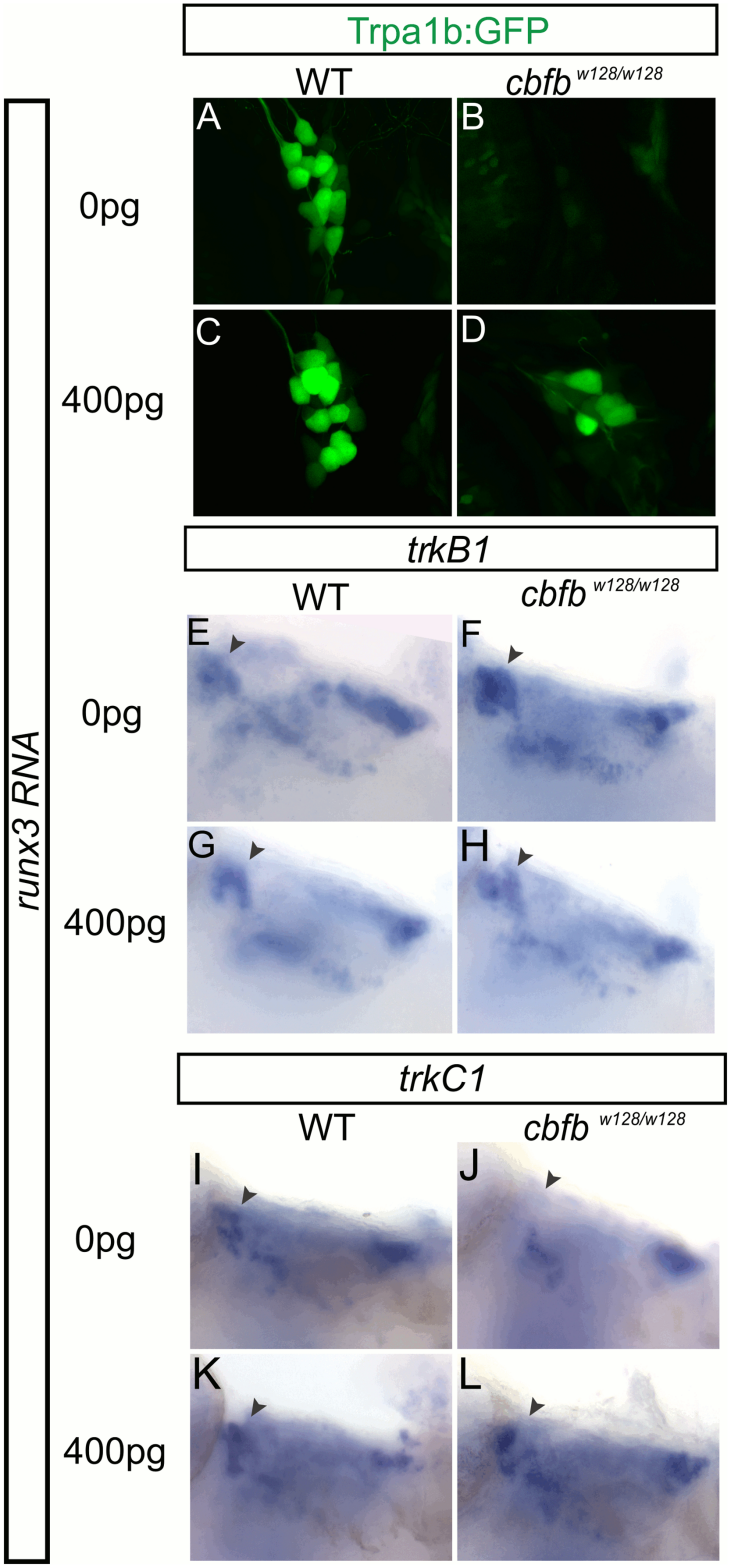
Runx3 overexpression rescues the *cbfb* mutant phenotypes in TG. A-D, Antibody staining for GFP of TG in *cbfb*^-/-^ *trpa1b*:*GFP* mutants injected with 0 or 400pg of *runx3* RNA at 3dpf. E-F *TrkB1* colormetric *in situ* hybridization staining for GFP in *cbfb*^*w128/w128*^; *trpa1b*:*GFP* mutants injected with 0 or 400pg of *runx3* RNA at 3dpf. I-L *TrkC1* colormetric *in situ* hybridization staining for GFP in *cbfb*^*w128/x128*^; *trpa1b*:*GFP* mutants injected with 0 or 400pg of *runx3* RNA at 3dpf.

We next examined whether overexpression of *runx* and/or *cbfb* could change the fates of somatosensory neurons resulting in ectopic Runx dependent gene expression in normally Runx-negative neurons. We monitored *trpa1b*:*GFP* expression in WT, *cbfb* or *runx3* mutant embryos after introduction of *runx1*, *runx3* or *cbfb* mRNA. In either the TG or in RBs, injection of *runx1*, *runx3*, or *cbfb* into WT embryos resulted in little change the number of neurons expressing GFP (Fig 10A and 10C; Table 10). Chromatin inaccessibility may prevent exogenous Runx/Cbfβ complex from promoting GFP expression in neurons that do not normally express it. To test this idea, we incubated embryos with the histone deacetylase inhibitor valproic acid in combination with *runx3* overexpression. Again we saw no increase in GFP-expressing neurons over WT levels (Table 10). This data indicates that Runx/Cbfβ signaling alone is not sufficient to drive *trpa1*:*GFP* expression in TG neurons.

**Table 10:** Rescue neuron counts.

TG	WT	*runx3*^*w144/w144*^		*cbfb*^*w128/w128*^	
		% rescued	Avg rescue	% rescued	Avg rescue
0pg	10.8±1.7 (6)	0% (10)		0% (9)	
50pg *runx1* RNA	10.5±0.8 (9)	37.5% (8)	2.0±0.7 (3)	0% (7)	
400pg *runx1* RNA	12.3±1.6 (4)	66.7% (3)	4.5±0.7 (2)	0% (4)	
50pg *runx3* RNA	11.7±1.0 (9)	71.4% (7)	9.2±2.7 (5)	0% (6)	
400pg *runx3* RNA	12.3±0.9 (11)	36.4% (11)	4.3±1.2 (4)	36.4% (11)	3.0±0.5 (4)
50pg *cbfb* RNA	12.8±1.6 (6)	0% (8)		20% (10)	3.0±0.0 (2)
250pg *cbfb* RNA	11.8±1.5 (8)	0% (8)		33.3% (9)	6.0±4.6 (3)
50pg *runx3* RNA 50pg *cbfb* RNA	13.6±0.6 (16)				
500uM VPA	8.4±0.8 (10)				
500uM VPA 50pg *runx3* RNA	8.5±0.5 (10)				
**RB**					
0pg	27.3±2.1 (6)	0% (10)		0% (9)	
50pg *runx1* RNA	37.8±1.3 (9)	75.0% (8)	6.2±1.7 (6)	0% (7)	
400pg *runx1* RNA	36.0±2.8 (4)	100% (3)	12.3±4.8 (3)	0% (4)	
50pg *runx3* RNA	33.8±2.9 (9)	85.7% (7)	16.2±2.4 (6)	0% (6)	
400pg *runx3* RNA	33.5±1.7 (11)	54.5% (11)	7.5±2.3 (6)	27.3% (11)	7.7±1.8 (3)
50pg *cbfb* RNA	26.5±2.6 (6)	12.5% (8)	5.0 (1)	0% (10)	
250pg *cbfb* RNA	29.1±2.4 (8)	25% (8)	7.0±1.4 (2)	33.3% (9)	26.3±5.5 (3)
50pg *runx3* RNA 50pg *cbfb* RNA	31.5±1.8 (16)				

% of fish with rescue and average number of *trpa1b*:*GFP* positive neurons in rescued fish at 3dpf in the *runx3*^*w144/w144*^ and *cbfb*^*w128/w128*^ mutants injected with *runx1*, *runx3* or *cbfb* RNA and/or incubated with valproic acid (VPA). Data represents mean±SEM (n).

We also tested whether Runx1 could compensate for loss of Runx3 in the TG, and whether overexpression of *runx3/runx1* could overcome the loss of *cbfb*. Each mRNA was able to partially rescue the loss of *trpa1*:*GFP* expression in the TG and RBs in corresponding mutants (Table 10). We saw little difference in the ability of *runx1* or *runx3* to rescue the *runx3* null mutant (Table 10), suggesting that they are functionally interchangeable and that differences in phenotype are due to spatial and temporal differences in expression. High concentrations (250pg) of *cbfb* RNA were unable to rescue *trpa1*:*GFP* expression in the TG of the *runx3* mutant (Table 10). Excess Cbfβ was able to partially rescue *GFP* expression in the RB population, likely due to its ability to interact with remaining Runx1 activity in these neurons (Table 10). We next sought to test whether Cbfβ is an obligate cofactor for Runx activity, given the similarities in phenotypes after *cbfb* and *runx* loss of function, by injecting *runx* mRNA into *cbfb* null mutants. High (400pg) but not low (50pg) concentrations of *runx3* RNA rescued GFP expression in *cbfb* null mutants in both TG and RB neurons (Fig 10B and 10D; Table 10). In *cbfb* null mutants that showed rescue of *trpa1*:*GFP* TG expression after injection of *runx3* RNA (400pg), we found rescue of *trkC1* expression in the TG of 78% of larvae (7/9) and suppression of *trkB1* expression in the TG of 38% of larvae (3/8) (Fig 10E–10L). Together these data indicate that Cbfβ is acting solely through its complex with Runx TFs to facilitate sensory neuron differentiation and that excess Runx can compensate for the loss of Cbfβ enhancement of Runx DNA binding affinity and/or Runx protein stability.

### *trkC1+* neurons switch fate to become *trkB1+* neurons

**Fig 11:**
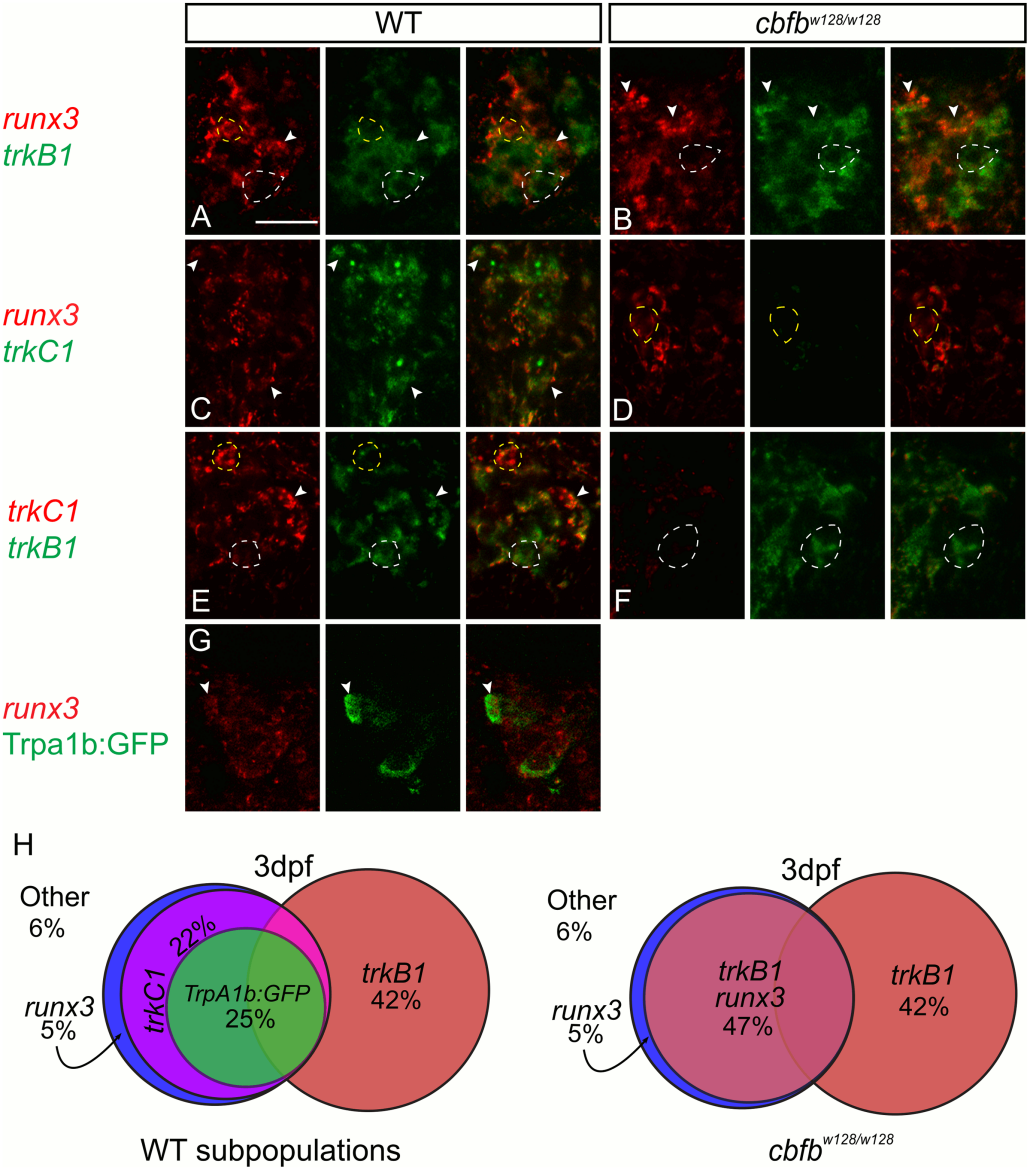
*trkC1+* neurons switch fate to become *trkB1+* neurons in the *cbfb* mutant zebrafish. **A-F**, Optical sections of double fluorescent *in situ* hybridization for *runx3* (red) and *trkB1* (green) **(A-B)**, *runx3* (red) and *trkC1* (green) **(C-D)**, and *trkC1* (red) and *trkB1* (green) **(E-F)** in wild-type and *cbfb* mutants. **G-I**, Optical sections of antibody staining of GFP (green) in conjunction with fluorescent *in situ* hybridization for *runx3* (**G**) in *trpa1b*:*GFP* fish at 3dpf. **H**, Representations of 3dpf wild-type and *cbfb* mutant somatosensory subpopulations as a percent of the whole TG. Dashed white line outline green cells; Dashed yellow lines outline red cells; Arrowhead indicates double positive cells. Scale bar: 20μm. Embryos per condition (n = 3–4). All error bars represent S.E.M.

We hypothesized that *trkC1*-expressing neurons adopted a *trkB1*+ cell fate in the absence of Runx function. Since expression of *runx3* is unchanged in *cbfb* mutants, we could examine the fates of *runx3+* cells in these animals. In WT fish, we found that the *runx3* population contained almost all cells that expressed the *trpa1b*:*GFP* transgene and *trkC1* mRNA (~96% of *trpa1b*:*GFP*+ cells express *runx3*; ~93% of *trkC1*+ cells express *runx3*; Fig 11C and 11G; Table 11). By contrast the *trkB1* population is largely separate from the *runx3*+/*trkC1*+/*trpA1b*:*GFP*+ population (~16% and ~21% of *trkB1*+ cells express *trkC1* and *runx3* respectively; Fig 11A and 11C; Table 11). In *cbfb* mutants, we found in nearly all *runx3*+ TG cells (86%) co-expressed *trkB1* (Fig 11A–11F; Table 11). These results, together with our earlier observations that showed no overall changes in TG neuron number or neuronal cell death, suggest that loss of Runx signaling results in *trkC1*-expressing neurons assuming a *trkB1*-expressing fate.

**Table 11:** Trigeminal neuron counts.

Double positive cells	WT	*cbfb*^*w128/w128*^
*runx3*+ *trkB1*+	7.3±0.4 (3)	25.3±1.8 (3)
*runx3*+ *trkC1*+	24.5±1.1 (4)	0.0±0.0 (3)
*trkB1*+ *trkC1*+	5.5±0.8 (4)	0.0±0.0 (3)
*runx3*+ *trpa1b*:*GFP*+	11.8±1.1 (4)	

Average number of double positive TG cells for *runx3+ trkB1+*, *runx3+ trkC1+*, *trkB1+ trkC1+*, *runx3+ trpa1b*:*GFP+*, *trkB1+ trpa1b*:*GFP+*, and *trkC1+ trpa1b*:*GFP+* at 3dpf in wild-type and *cbfb*^*w128*^ mutants. Data represents mean±SEM (n).

**Fig 12:**
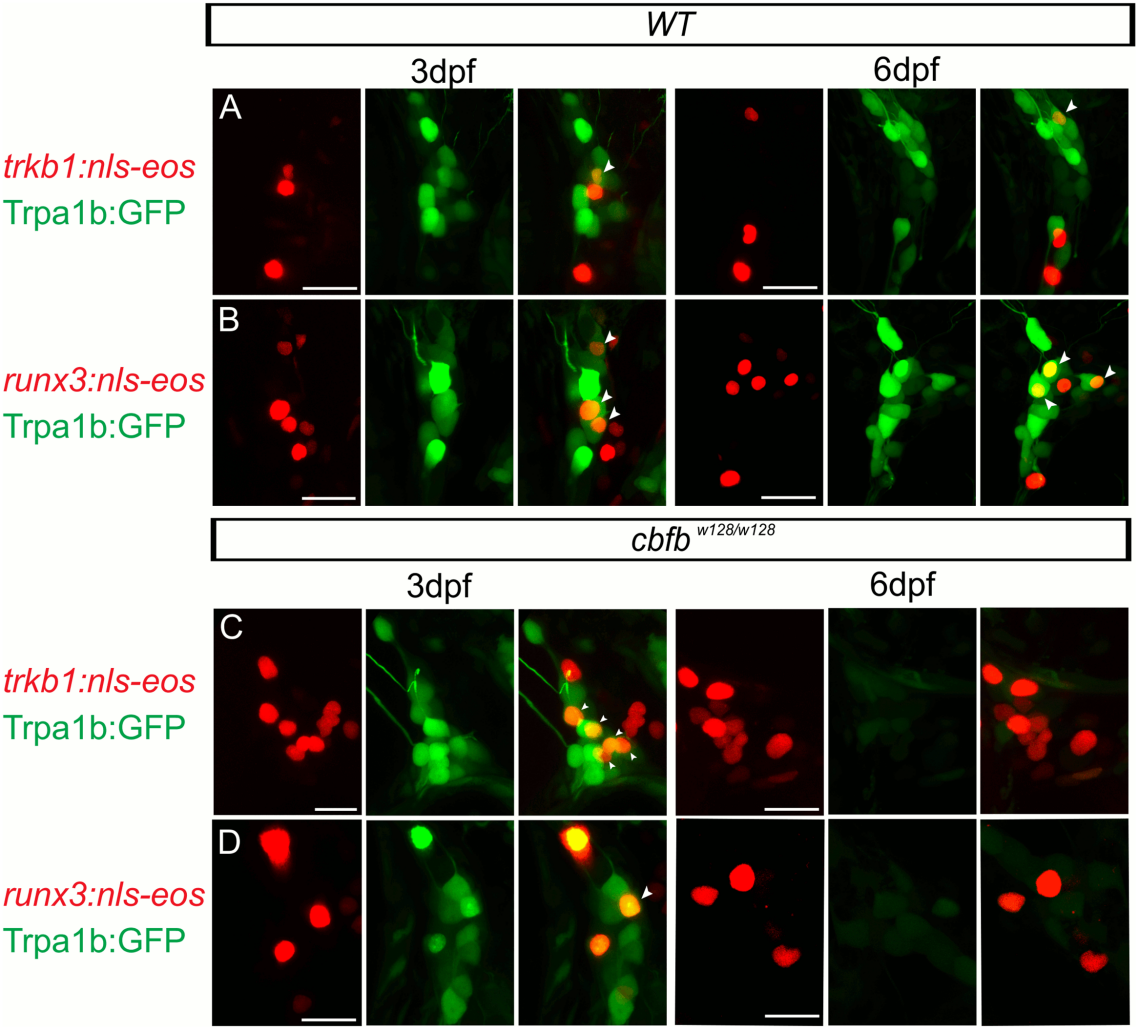
Scatter labeled *trkB1* and *runx3* neurons persist in *cbfb*^*w128/w128*^; *trpa1b*:*GFP* embryos. (**A-D**) Maximum intensity projections of transiently expressed nls-Eos in *trpA1b*:*GFP* fish at 3dpf and 6dpf. **(A)** *trkb1*:*nls-Eos* in a WT *trpa1b*:*GFP* embryo, **(B)** *runx3*:*nls-Eos* in a WT *trpa1b*:*GFP* embryo, **(C)** *trkB1*:*nls-Eos* in a in *cbfb*^*w128/w128*^; *trpa1b*:*GFP* embryo, **(D)** *runx3*:*nls-Eos* in a in *cbfb*^*w128/w128*^; *trpa1b*:*GFP* embryo. Arrowhead indicates double positive nls-Eos/GFP TG neurons. Scale bar: 20μm.

To test directly whether cells changed fate, we labeled cells with the photoconvertible protein Eos. We first examined the time course of expression loss of *trpa1b*:*GFP* using high-resolution confocal microscopy. With this methodology, we observed GFP expression in *cbfb* mutants at 3dpf, when *trkC1* and *trpa1b* expression are absent as measured by *in situ* hybridization (see Figs 5 and 6) However, GFP was absent by 6dpf (S8 Fig). The ability to visualize these neurons at 3dpf indicated that these neurons were still present and had not been replaced in *cbfb* mutants with a new *runx3+* neuronal subtype. We then performed scatter-labeling of TG neurons by CRISPR-mediated insertion of nls-Eos into the *trkB1* or *runx3* promoter region of WT and *cbfb* mutants transgenic for *trpa1b*:*GFP*. At 3dpf we photoconverted nls-Eos and imaged the TG to observe residual GFP to identify double positive neurons (Fig 12). We then re-imaged the same animals at 6 dpf when GFP was absent. In all animals imaged, all photoconverted runx3:nls-Eos (WT, 15/15 TG neurons, n = 4 animals; cbfb mutants, 12/12 TG neurons, n = 4 animals) and trkB:nls-Eos (WT, 7/8 TG neurons, n = 2 animals; cbfb mutants, 11/11 TG neurons, n = 2 animals) neurons were still present at 6dpf including those that were formally double positive neurons in *cbfb* mutants. While these results are consistent with survival at the time points measured, we cannot rule out that these neurons may be lost at later stages. In sum, these data support our conclusion that *runx3+* neurons that would normally express *trkC1* in WT animals assume a *trkB1*-expressing cell fate in Runx signaling mutants.

## Discussion

In this study, we set out to define the roles that zebrafish Runx/Cbfβ complexes play in refining early larval somatosensory cell fate specification. We first examined expression of neurotrophin receptors that in mammals define distinct somatosensory populations. It is inherently difficult to compare potentially dynamic developmental expression patterns across species due to the temporal differences in development and differences in the time points examined. In most terrestrial vertebrates the neurotrophin receptors TrkA, TrkB and TrkC are required respectively for the survival and specification of distinct classes of nociceptive, mechanoceptive and proprioceptive (DRG) or mechanoceptive (TG) somatosenosory neurons. In contrast, we found in larval zebrafish that the neurotrophin receptor orthologs *trkA*, *trkB1*, and *trkC1* were expressed in distinct and overlapping patterns in nociceptive neurons as determined by the coexpression of the nociceptive ion channels *trpv1* and *trpa1b*. Collectively these neurons account for nearly 90% of the TG at 3dpf with the remaining neurons not expressing any of these markers. These findings are in line with our previous work that showed that all early born TG neurons expressed nociceptive markers [13]. It is possible that zebrafish neurotrophin receptors segregate into different populations of somatosensory neurons or take on additional roles similar to terrestrial vertebrates at later developmental stages.

To test the roles of Runx TFs in regulating somatosensory neuron specification, we obtained a *runx1* mutant and generated loss of function mutations in *runx3* and *cbfb* by genome editing. In contrast to mammals in which Runx1 and Runx3 have distinct functions regulating nociceptors and proprioceptors, we found that zebrafish *runx1* and *runx3* both regulate nociceptors development. Both *runx1* and *runx3* play an overlapping role to influence RB nociceptor development in the body, while in the TG, only *runx3* has this function. Analogous to mammalian Runx1, zebrafish Runx3 controls the expression of the sensory receptors *trpA1b* and *piezo2b*. However while mammalian Runx1 additionally acts to suppress *trkA* and *cgrp* expression and promote *ret*, *p2x3* and *trpv1* expression, loss of zebrafish *runx3* in the TG has no apparent effect on these markers at the time points examined.

As for mammals, we found that loss of zebrafish *runx3* results in loss of *trkC1* expression and an increase in *trkB1* expression, despite the fact that all these genes are expressed in nociceptors in early larval zebrafish and not mechanoreceptors/proprioceptors as in mammals. Taken together these results demonstrate that the core Runx regulatory program is conserved amongst larval zebrafish and mammals. Future experiments examining loss of function of zebrafish neurotrophin genes will be necessary to determine to what degree the roles of neurotrophin receptors in the specification of somatosensory cell fate have diverged.

Regulation of expression of *runx* genes differed depending on what population of somatosensory neurons they were expressed. Runx/Cbfβ signaling in RB neurons was required for maintaining *runx* expression as *runx1* and *runx3* expression was lost in *cbfb* null mutants and loss of *runx1* function led to loss of *runx3* expression and to a lesser extent vice versa. This suggests that a primary role of Runx1 in RB neurons is to facilitate *runx3* expression. These results are consistent with findings that Runx proteins have been shown to regulate their own promoters [24]. In the TG, loss of *cbfb* did not alter *runx3* expression at the timepoints examined, suggesting that either Runx3 can maintain its own expression in the absence of *cbfb* or that an idependent mechanism controls *runx3* expression. Loss of *runx* had no effect on *cbfb* expression in either the TG or RB neurons. This result is consistent with studies in mouse DRG where *Runx* and *Cbfb* gene expression were shown to be regulated independently [25].

Cbfβ can act as an obligate cofactor for Runx activity in some tissues while acting to enhance Runx function in others. Cbfβ enhances Runx binding to DNA 5 to-10-fold as well as promotes Runx protein stability. For example, in mouse, a conditional deletion of Runx1 and a conditional rescue of Cbfβ indicated that the formation of hematopoietic stem cells (HSCs) and erythroid/myeloid progenitors required both Runx1 and Cbfβ [26,27]. By contrast in zebrafish, Cbfβ mutants are able to form Runx1-dependent HSCs [28]. In addition, Runx2-dependent intramembranous and endochondral ossification can develop further in the absence of Cbfβ function than they do in Runx2^-/-^ mice [29]. This suggests that in some cases Cbfβ may act to refine Runx function but is not essential for all Runx-dependent activity. A recent study in mice found that conditional knockouts of Runx1 and Cbfβ in the DRG, had identical somatosensory neuron cell fate specification defects, suggesting that in this context Cbfβ serves as an obligate cofactor of Runx1 [25]. Howerver, loss of Cbfβ in the DRG resulted in the loss of Runx1 protein but not *runx1* mRNA, so it was not possible to measure the result of Runx transcriptional activity in the absence of Cbfβ [25].

We found that in zebrafish Cbfβ serves as an obligate cofactor of Runx3 such that all somatosensory defects caused by *runx3* deletion were phenocopied by *cbfb* deletion. However expression of high (but not low) levels of *runx3* mRNA was able to rescue *trpa1b*:*GFP* expression and *trkC1* expression while suppressing *trkB1* expression in *cbfb* mutants, suggesting that Runx can function in the absence of Cbfβ. In wild-type fish, neither *runx3* and/or *cbfb* RNA could drive etopic expression of *trpa1b*:*GFP*. Additionally, although *runx* and *cbfb* are also expressed in other cranial ganglia in addition to the TG, they do not affect the expression of *trkC1* or *trpa1b* in these structures. These results suggest that the role of the Runx/Cbfβ interaction in managing the expression of these genes is highly specific to somatosensory neurons and may require the coexpression of other factors to mediate its effect.

Our data supports the investigation of the genetic determinants of somatososensory neuron development/function in early born larval zebrafish. We have revealed a neurotrophin receptor code in larval zebrafish that is substantially divergent from that of terrestrial vertebrates, yet shown that the developmental program that gives rise to somatosensory neuron diversity remains largely intact.

Our results suggest that in terrestrial vertebrates Runx1 and Runx3 gained additional distinct roles by subfunctionalization, with Runx1 taking over most functions in nociceptors and Runx3 acquiring additional prominent roles in proprioceptors (DRG) or mechanoreceptor (TG) specification. Terrestrial vertebrates have a much larger and well-defined set of proprioceptive neurons, presumably gained with the need for postural somatosensory feedback that occurred as a consequence of the acquisition of tetrapod limbs and the move to land. It is not clear however if teleost fish require the light touch mechanoreceptors and proprioceptors associated respectively with TrkB and TrkC in terrestrial vertebrates [30]. The need for these sensations in terrestrial vertebrates may have necessitated the ceding of nociceptor specification to TrkA and the repurposing of TrkB and TrkC to promote mechanosensory and proprioceptive neuronal fates. The dominance of nociceptive markers in larval zebrafish suggests that early nociceptive development is critical for survival of the free-swimming larvae and may therefore take precedence over other somatosensory modalities.

## Materials and methods

### Ethics statement

Experiments using zebrafish were performed under the University of Washington Institutional Animal Care and Use Committee protocols #4216–02 (approved on 9/16/2016). The University of Washington Institutional Animal Care and Use Committee (IACUC) follow the guidelines of the Office of Laboratory Animal Welfare and set its policies according to The Guide for the Care and Use of Laboratory Animals. The University of Washington maintains full accreditation from the Association for Assessment and Accreditation of Laboratory Animal Care (AAALAC) and has letters of assurance on file with OLAW. The IACUC routinely evaluates the University of Washington animal facilities and programs to assure compliance with federal, state, local, and institution laws, regulations, and policies. The OLAW Assurance number is DL16-00292.

### Zebrafish

Zebrafish were maintained at 28.5°C on a 14h/10h light/dark cycle following established methods. Embryos were maintained in E2 medium, and staged according to the standard manual [31]. *runx1*^*W84X*^ mutants were obtained from the Liu laboratory (National Institutes of Health, Bethesda, MD, USA) [16]. A subset of the trigeminal was identified using *TgBAC(trpa1b*:*EGFP*)^*a128TG*^ and *Tg(isl1*:*Gal4-VP16*,*14xUAS*:*Kaede*)^*a128*^, referred to as *trpa1b*:*GFP* and *Isl1SS*:*Kaede*, from the Schier laboratory (Harvard University, Cambridge, MA, USA) [3], and Tg(*p2rx3b*:*EGFP*)^sl1Tg^, referred to as *p2x3b*:*eGFP*, from the Voigt laboratory (Saint Louis University School of Medicine, St. Louis, MO, USA) [23].

### Generation of the zebrafish *cbfb* and *runx3* truncation mutations

All mutations were generated the AB strain of zebrafish and prior to analysis each genetic mutant was backcrossed for at least two generations into the AB background.

### TALEN target site selection and assembly

Transcription activator-like effector nucleases (TALENs) were used to generate a mutation in zebrafish *cbfb*. TALENs were assembled using the Golden Gate assembly protocol and library [17]. TALE binding sites in exon 2,of the *cbfb* genomic sequence, 5′- TTAAATACACCGGTTTCCGC-3′ and 5′-TTCTGGAAGCGCGCCTGCCT-3′, were identified using TALE-NT 2.0 [32].

### CRISPR target site selection and assembly

sgRNAs were designed using http://crispr.mit.edu. We used a two-oligo PCR method to make the template DNA [33]. A scaffold oligo containing the Cas9 recognition loop and an oligo with a T7 binding site, the *runx3* sgRNA sequence, and homology to scaffold oligo were synthesized. The scaffold oligo sequence was 5′-GATCCGCACCGACTCGGTGCCACTTTTTCAAGTTGATAACGGACTAGCCTTAT TTTAACTTGCTATTTCTAGCTCTAAAAC-3′. The *runx3* sgRNA oligo sequence was 5′-AATTAATACGACTCACTATA(GTGCAACAAAACCCTTCCCG)GTTTTAGAGCTAGAAATAGC-3′; the target in exon 3 is indicated in parentheses.

### mRNA synthesis and microinjection of zebrafish embryos

TALEN expression vectors were linearized with SmaI and transcribed *in vitro* using the mMessage mMachine T7 and Poly(A) Tailing Kit (ThermoFisher). The pT3TS-nCas9n plasmid (Addgene), linearized using XbaI and purified [18], was used in an *in vitro* transcription reaction (T3 mMessage mMachine, ThermoFisher). The *runx3* guide RNA was synthesized using the MegaScript T7 Kit (ThermoFisher). RNA products were cleaned by phenol-chloroform and isopropanol precipitation. The TALEN mixture containing equal amounts of each mRNA (400 pg each) was injected into one-cell stage AB strain zebrafish embryos. The CRISPR/Cas9 injection contained 300pg of Cas9 mRNA and 50ng of *runx3* gRNA and was also injected into one-cell stage zebrafish embryos. Injected embryos were raised to adulthood, outcrossed, and gDNA isolated from F1 embryos to identify mutants.

### Genomic DNA Isolation

Individual embryos were processed as previously described [34]. Embryos were incubated in 1x base solution from a 50x stock (1.25 M NaOH, 10mM EDTA pH12) at 95°C for 30 min in 25μl and then a 2x neutralization solution from a 50x solution (2M Tris-HCl pH5) was added.

### High Resolution Melt (HRM) curve analysis

Primers for identifying the *cbfb* mutation were 5′- AACACTCTTCTGTGCCTTTTTCATCC -3′ and 5′- TGAGGTGCGTGTACTCACTATCTCTG -3′. Primers for the *runx3* mutation were 5′- CCAAACTTTCTCTGCTCGGTCCT -3′ and 5′- GAGCGCGAGTTCTGTTTGTAGC -3′. HRM mix contained 400μl 5x Gotaq buffer (Promega), 40μl 10mM dNTPs, 120μl 25mM Mgcl2, 100μl DMSO (Sigma), 100μl 20x EvaGreen (Biotium), 60μl Taq, and up to 1000μl water. PCR reactions contained 0.5μl of each primer (10μM), 10μl of HRM mix, 1μl of gDNA, and water up to 20μl. PCR was performed in a BioRad CFX Connect, using 96 well plates (BioRad cat. No. HSP9601). PCR reaction protocol was 95°C for 2 min, then 40 cycles of 95°C for 45 sec, 60°C for 30 sec, and 72°C for 30 sec, followed by 95°C for 30 sec and 60°C for 1 min. Melting curves were generated over a 65–95°C range. Curves were analyzed with the BioRad Precision Melt Analysis Software to identify mutations. Sequencing identified *cbfb*^*w128*^ as a 4bp deletion (nt109-112 (CACG)) and *runx3*^*w144*^ as a 1bp deletion (nt247 (C)), which both predict an early truncation (S1 Fig).

### DNA constructs

#### *In situ* probe constructs

A clone for Ret (Clone ID: 9038324) was purchased from Open Biosystems. *scn1a* and *piezo2b* were cloned from total RNA extracted from 72hpf zebrafish embryos. *scn1a* and *piezo2b* cDNA was amplified by performing reverse transcription PCR with Superscript II (ThermoFisher) using primers (F: 5’- gctagaattcTTTACTCCGCCAGGACCT-3’; R: 5’- gctagtcgacTAAAGCGTCCCACACAG -3’) and (F: 5’- gatcgaattcGTCTTTCTGATCTGGTCCT -3’; R: 5’- gatagtcgacTGGT GCTCTCCTGTTTG -3’) respectively. *scn1a* and *piezo1b* cDNA were cloned into pBluescript SK+ (Stratagene) for *in situ* hybridization with EcoRI and SalI.

### mRNA injection

Full length *cbfb*, *runx1*, and *runx3* were cloned from total RNA extracted from 72hpf zebrafish embryos. *cbfb*, *runx1*, and *runx3* cDNA were amplified by performing reverse transcription PCR with Superscript II (Invitrogen) using primers (F: 5’- GATAGAATTCATGCCTCGGGTGGTCC -3’; R: 5’-GATAGTCGACCTAGCGCATCTTGTGATCATCAGT-3’), (F: 5’- taatacgactcactatagggATGGTTTTTCTTTGGGACGCC-3’; R: 5’- TCAGTATGGCCTCCAGACGG -3’), and (F: 5’-GCTAGGTACC ATGCATATTCCCGTAGACC-3’; R: 5’-GCGCGAATTCtttctaaatcttagtacggc-3’) respectively. *cbfb* and *runx3* coding sequences were TA cloned into pCR2.1 (ThermoFisher), linearized with KpnI and transcribed *in vitro* using the mMessage mMachine T7 and Poly(A) Tailing Kit to generate capped, polyadenylated mRNA. The *runx1* coding sequence was purified and transcribed *in vitro* using the mMessage mMachine T7 and Poly(A) Tailing Kit to generate capped, polyadenylated mRNA. A range of mRNA concentrations was injected into embryos derived from a cross between *trpa1b*:*GFP/runx3*^*+/w144*^ and *Tg(Trpa1b*:*GFP)/cbfb*^*+/w128*^ parents. Data were analyzed using two-factor ANOVA.

### Whole mount *in situ* hybridization and antibody staining

Digoxigenin (DIG) labeled riboprobes for *runx1*, *cbfb* [28], *cgrp* [3], *trkA*, *trkB1*, *trkC1* [35], *trpv1*, and *trpa1b* [13] and fluorescein (FLR) labeled riboprobes for *trkB1* and *trkC1* were generated as previously described. Full length (2.1kb) *trkA* was amplified using primers (F: 5’- ATGGCTGACCATAGGGTGGCC-3’ and R: 5’- TAATACGACTCACTATAGGG CTACTCCAGGATGTCCAGGTAGAC-3’) and transcribed with T7 polymerase (ThermoFisher) to generate DIG-labeled riboprobe. An 870bp (colormetric) or 450bp (fluorescence) *runx3* fragment was amplified using primers (F: 5’- gcatattcccgtagacccga-3’ and R: 5’- gatcTAATACGACTCACTATAGGGCGGA GTATGTGAAGTG-3’; F: 5’-agccacttcacatactccgc-3’ and R: 5’-gatcTAATACGACTCACTAT AGGGttagtacggcctccag-3’) respectively and transcribed with T7 polymerase. *ret* was linearized with NotI and transcribed with T3 polymerase (ThermoFisher). *scn1a* and *piezo2b* were linearized with EcoRI and transcribed with T7 polymerase.

*In situ* hybridization was carried out as previously described [36,37]. In brief, embryos were hybridized with DIG-labeled or FLR-labeled RNA probes overnight at 55°C followed by stringent washes. Samples were incubated with anti-DIG Biotin-conjugated Fab fragments (Roche, 1:1000 DIG, 1:500 FLR) and then incubated with Cy3- or FITC-tyramide (PerkinElmer). Embryos were stained with mouse anti-Elavl antibody (ThermoFisher HuC+HuD antibody 16A11, 1:1000) to identify trigeminal sensory neurons and/or rabbit anti-GFP (Invitrogen, 1:1000) to identify a subset of trigeminal sensory neurons in *trpa1b*:*GFP* fish as previously described and imaged by confocal microscopy [13]. *Isl1SS*:*Kaede* fish were stained with rabbit anti-Kaede antibody (MBL International, 1:1000). Active caspase-3 staining was used to identify TG neurons undergoing apoptosis with rabbit anti-active Caspase 3 (ThermoFisher bdb559565). In double *in situ* hybridization experiments, double positive cells were identified as consistently as possible by shape. Colocalization of Elavl, GFP, or an *in situ* probe with an *in situ* probe was analyzed by confocal imaging in single optical planes. Data were analyzed using ANOVA.

### Behavioral analysis

Larvae were raised on a 14/10 h light/dark cycle at 28.5°C. At 5dpf, individual larvae were placed in single wells on a 100 M 96-well mesh plate (MANM 100 10; Millipore). The base plate, into which the mesh plate was inserted, was constructed from .002” aluminum Shim in a can (ASTM–B– 209; Shopaid), which had been scrubbed. Base plates were thoroughly washed and soaked in distilled water before use. Two base plates filled with embryo medium were placed on each side of a dual solid-state heat/cool plate (AHP-12000CP; Teca) set to control and test temperatures, with an intervening film of water to facilitate temperature transfer. Larvae were loaded into each well of the mesh plate placed on the control side with a cut pipette. The mesh plate was then transferred from control to test temperature and larval movement videotaped for 4 minutes. To assay chemical responses, larval movement was recorded for 4 min after the mesh plate was placed into the lid of a 24 multiwell tissue culture plate (353047; BD Labware) containing allyl isothiocyanate (AITC; mustard oil; Sigma-Aldrich 377430; diluted in 1% DMSO). Behavioral responses were recorded using a Canon high definition digital video camcorder with a frame rate of 60 fps. The locomotor activity of each larva was analyzed with EthoVision XT locomotion tracking software (Noldus Information Technology, Inc.). Data were analyzed using ANOVA.

### Tail deflection behavior

At 3dpf, the larval zebrafish offspring of a *runx1*^*+/W84X*^ cross were imbedded in 1.5% agarose at the bottom of individual wells filled with 10mL of E2 medium. The agarose surrounding the tail of each fish was cut away allowing the tail to float freely. These fish were then exposed to either 150μM AITC (Sigma-Aldrich) in 1% DMSO (Sigma-Aldrich), 1% DMSO vehicle, or touch with 0.018cm diameter fishing line (Maxima). All solutions contained phenol red (Sigma-Aldrich) for visualization and were applied in a 50ms pulse by a Picosprizter II microinjection apparatus (General Valve Corporation). The behavioral response of each fish was recorded using a Canon high definition digital video camcorder with a frame rate of 60 fps and were manually scored based on the number of tail deflections. Following behavioral analysis animals were genotyped as described. Statistical significances was determined using ANOVA.

### CRISPR/Cas9 mediated scatter labeling of TG neurons

Transient transgenic labeling of trigeminal neurons was performed as previously described [38]. In short, single cell embryos are injected with Cas9 protein, a reporter containing plasmid, a guide RNA (gRNA) targeting the endogenous promoter of trkB1 or runx3, and a gRNA targeting the injected plasmid. mRuby3 (pKanCMV-mClover3-mRuby3 was a gift from Michael Lin (Addgene plasmid # 74252)) and nls-Eos reporter plasmids were generated by cloning into the XbaI/BamHI sites of pbsk-Mbait-Hsp-GFP (gift from Shin-ichi Higashijima)[39]. The following gRNA sequences were used: mBait (GGCTGCTGCGGTTCCAGAGG), runx3 (GGGTTTAAGCGACCAATCAG), and trkB1 (GGTGTGTTTGCTGCTTCGTG).

At 3dpf injected embryos were photoconverted using a DAPI filter and screened for nls-Eos expression. Embryos expressing nls-Eos in the TG were anesthetized with Mesab, mounted in 2% agarose, and imaged on a Zeiss LSM 880 confocal microscope. They were then returned to standard rearing conditions, before imaging at 6dpf.

## Supporting information

S1 Fig CRISPR mediated knock-in of mRuby into the trkB1 promoter recapitulates in- situ results. **A-C**, Maximum intensity projection of a single TG from a 3dpf TrpA1:GFP (green) embryo transiently expressing mRuby from the trkB1(red) promoter. Arrowhead indicates double positive cells. Scale bar: 20μm.
(TIFF)

S2 Fig *runx1*^*w84x*^, *runx3*^*w144*^, and *cbfb*^*w128*^ mutations. **A**. Location of mutations in exons. **B**. Nucleotide location of mutations and predicted early termination stop codon. **C**. Location of predicted early stop in protein structure.
(TIFF)

S3 Fig Runx1 and Runx3 act cooperatively to promote *runx* and *trk* receptor expression in RB neurons. **A-T**, Colormetric *in situ* hybridization for *runx1* **(A-D)**, *runx3* **(E-H)**, *trkB1* **(I-L)**, *trkC1* **(M-P)** *trpa1b* **(Q-T)** in RB neurons of WT, *runx1*^*+/W84X*^*/runx3*^*+/w144*^, *runx1*^*+/W84X*^*/cbfb*^*+/w128*^ *and runx3*^*+/w144*^*/cbfb*^*+/w128*^ *double* heterozygous mutants at 18s (runx1) and 24hpf (runx3, *trkB1*, *trkC1*, and *trpa1b*). **U**, Quantification of marker gene expression as % WT at 18s or 24hpf. Arrow, RBs; Scale bar: 100 μm. Embryos per condition (4–15). *p<0.05, **p<0.01, ***p<0.001. All error bars represent S.E.M.
(TIFF)

S4 Fig Loss of *runx1* does not affect *runx3* or Trpa1b:GFP expression in the TG. **A-B**, Colormetric *in situ* hybridization for *runx3* in WT **(A)** and *runx1*^*W84X/W84X*^ null mutants **(B). C-D**, Trpa1:GFP expression in WT **(C)** and *runx1*^*W84X/W84X*^ null mutants **(D)**.
(TIFF)

S5 Fig Loss of *runx* or *cbfb* expression does not affect neuronal number. **A-G**, Antibody staining for Elavl at 24hpf and 3dpf in the *runx1*^*W84X/W84X*^, *runx3*^*w144/w144*^, and *cbfb*^*w128/w128*^ mutants. **H-I**, Quantification of Elavl expression in the RB population at 24hpf and the TG population at 24hpf and 3dpf. Scale bar: 20μm. Embryos per condition (n = 3–26). All error bars represent S.E.M.
(TIFF)

S6 Fig Loss of *runx* or *cbfb* expression did not affect anti-active caspase3 staining. **A-P**, Maximum intensity projections of antibody staining for HuC (green) and active caspase3 (red) at 1dpf **(A-D)**, 1.5dpf **(E-H)**, 2dpf **(I-L)**, or 3dpf **(M-P)** Scale bar: 20μm.
(TIFF)

S7 Fig Loss of *runx* or *cbfb* expression did not affect the expression of nociceptive subtype markers. **A-I**, Colormetric *in situ* hybridization for *cgrp* **(A-C)**, *ret* **(D-F)**, and *scn1α* **(G-I)** in the *runx3*^*w144/w144*^ and *cbfb*^*w128/w128*^ mutants focusing on the TG at 24hpf and 3dpf. **J-M**, Antibody staining of Kaede (red) in *cbfb*^*w128/w128*^; Isl1SS:Kaede mutants and of GFP (green) in *cbfb*^*w128/w128*^; P2X3.2:eGFP mutants at 24hpf and 3dpf. **N-O**, Quantification of Kaede+ and GFP+ cells in the TG at 24hpf and 3dpf. **P- S**, Colormetric *in situ* hybridization for *scn1α* in the *runx1*^*W84X/W84X*^, *runx3*^*w144/w144*^ and *cbfb*^*w128/w128*^ mutants focusing on the RBs at 24hpf and 3dpf. **T**, Quantification of marker gene expression as %WT in RBs at 24hpf. Arrow, RBs; Arrowhead, TG. Scale bar: A-M 100 μm, N-Q 20μm. Embryos per condition (n = 3–11). All error bars represent S.E.M.
(TIFF)

S8 Fig Residual GFP allows for visualization of TrpA1b fated neurons in the in the *cbfb*^*w128/w128*^ mutant. **A-H**. Maximum intensity projections of TrpA1b:GFP expression from the same TG in a WT **(A- D)** or *cbfb*^*w128/w128*^ **(E-H)** embryo over five days. Scale bar: 20μm.
(TIFF)

S1 Video WT larvae do not respond to puff of vehicle control onto tail. (MP4)

S2 Video *runx1*^*w84x*^ mutant larvae do not respond to puff of vehicle control onto tail. (MP4)

S3 Video WT larvae tail deflect in response to puff of AITC onto tail. (MP4)

S4 Video *runx1*^*w84x*^ mutant larvae do not respond to puff of AITC onto tail. (MP4)

S5 Video WT larvae tail deflect in response to touch onto tail. (MP4)

S6 Video *runx1*^*w84x*^ mutant larvae tail deflect in response to touch onto tail. (MP4)
